# Experimental ^1^H, ^13^C and T_1_ NMR Studies of Graphene Oxide Interactions with 2-Fluorophenylacetic Acid as a Fluorinated Cathinone Model Supported by Molecular Modelling

**DOI:** 10.3390/molecules31111801

**Published:** 2026-05-24

**Authors:** Natalina Makieieva, Michał Jewgiński, Artur Małolepszy, Teobald Kupka

**Affiliations:** 1 Faculty of Chemistry and Pharmacy, University of Opole, 48, Oleska Street, 45-052 Opole, Poland; 2 Department of Bioorganic Chemistry, Faculty of Chemistry, Wrocław University of Science and Technology, 27, Wybrzeże Wyspiańskiego Street, 50-370 Wrocław, Poland; michal.jewginski@pwr.edu.pl; 3 Faculty of Chemical and Process Engineering, Warsaw University of Technology, 1, Waryńskiego Street, 00-645 Warsaw, Poland; artur.malolepszy@pw.edu.pl

**Keywords:** cathinone, 2-fluorophenylacetic acid, graphene oxide, complex stability, NMR, DFT

## Abstract

Cathinone and its synthetic derivatives are among the most popular drugs worldwide. However, the literature provides data on the medicinal and cytotoxic potential of some of these compounds. These data are extremely limited due to the need to obtain additional permits for laboratory studies. Consequently, the therapeutic potential of cathinones may not have been fully explored. Furthermore, the literature provides data on the reduction or reversal of undesirable biological properties of drugs encapsulated in a bio-compatible carrier and administered through targeted therapy. The current study presents preliminary theoretical and experimental tests for further research on target cathinone–graphene–oxide complexes. A non-psychotropic cathinone model—o-fluorophenylacetic acid—was used. The NMR properties (chemical shifts, spin–spin coupling constants, and T_1_ relaxation times) of graphene oxide–F-derivative complexes were measured at an acidic and neutral pH. To analyze the structure and stability of the possible complexes in different environments, molecular modelling was performed with simplified graphene oxide models using density functional theory. Experimental data were compared with theoretical values, and the most stable structures that may account for the observed spectral properties of the studied complexes were presented. The obtained data indicate a stronger tendency towards the formation and stabilization of GO-2-fluorophenylacetic acid complexes in a neutral environment.

## 1. Introduction

Cathinone and its synthetic derivatives are among the most popular groups of narcotics in the world. Such big interest is driven by a wide range of their psychotropic prop-erties with varying potency, as well as by low cost and ease of drugs synthesis [1,2,3]. Consequently, new derivatives are constantly appearing on the drug market, and increasing attention is being paid to them in the scientific literature [4]. In addition to cathinone’s psychotropic properties [1] and the validation of analytical methods for its identification and classification from various matrices [5,6,7], attention is also paid to its potential medicinal properties. For example, there are extensive literature data on bupropion, the only registered antidepressant and smoking-cessation agent from the cathinone group [8,9]. Limited data can be found on the potential of some other derivatives for the treatment of obesity and depression [10,11]. Such limitations are due to the complexity of conducting experimental studies of the biological properties of cathinones. Studies on drugs require special permits. Furthermore, the psychotropic side effects often complicate the planning and conducting of analyses of other potential medical properties. For example, the cytostatic activity of cathinones has been described in the literature [12]. These cytotoxic properties were studied on a number of cancer cell lines [13,14,15,16]. Potential antitumour effects have been observed with the modification of cathinone molecules in different positions [14]. Furthermore, an increase in cytostatic activity has been noted in some studies with the development of tumor carcinogenesis [17,18]. In-depth studies on these properties could open prospects for the development of new anticancer drugs based on cathinone’s molecular skeleton. However, the incomplete understanding of the structure–psychotropic activity correlation is a significant limitation for these studies [14]. For this reason, it is necessary to pay attention to both the structure of the studied cathinones and the methods of their administration. One popular and widely described method of transporting drugs with potentially dangerous side effects is targeted delivery via drug delivery systems (DDS) [19]. This kind of administration allows for the medicine to be transported to the molecular target in the maximum possible quantity and reduces or avoids its undesirable properties, as the drug is shielded by a protective carrier until it is released near the target [20]. The literature contains data on DDS with both classical cytostatics [21,22] and potential antitumor drugs [23,24]. Both synthetic and natural polymers are often used as carriers in these systems [25,26,27]. Evidence has also emerged regarding the possibility of using ordered carbon nanostructures as anticancer drug carriers [28,29,30,31,32]. Therefore, it could be assumed that delivering cathinones in a protective carrier could potentially reduce or eliminate their psychotropic effects on the body and fully utilize their cytotoxic potential. However, the scientific literature lacks systematic experimental studies on the interactions of cathinones with drug carriers of different structures and origins. The current study is intended to serve as an introduction to a further in-depth exploration of the cathinones interactions with ordered carbon nanostructures under varying pH conditions. In our previous work [33], we conducted a systematic theoretical study of the interactions of a number of cathinone derivatives with carbon nanotubes. In silico studies were performed in simplified environments with an acidic and neutral pH. The results demonstrated the potential for stronger intermolecular interactions in a neutral environment. This may indicate the possibility of developing complexes, based on cathinones and carbon nanostructures, that exhibit maximum stability in the neutral environment of healthy tissue and will decompose in a controlled manner in the acidic environment of tumours. Based on this hypothesis, we decided to conduct preliminary experimental studies on complexes using graphene oxide (GO) as a carrier in neutral and acidic environments. Since no permits were obtained for experimental studies with narcotic substances, we used a model of cathinones–o-fluorophenylacetic acid. This compound was chosen as a model of fluorinated cathinones as the literature data indicate an increase in cytostatic properties and a decrease in psychotropic properties upon halogenation of the benzene ring of cathinone [34]. The aim of this study was to identify changes in the ^1^H and ^13^C NMR spectra (chemical shifts and spin–spin coupling constants), as well as the proton T_1_ relaxation times, in GO complexes with the studied 2-fluorine derivative at neutral and acidic pHs. The results were compared with in silico data, obtained using density functional theory (DFT), to identify the structures of most stable complexes and to examine potential geometric parameters leading to the observed changes in the experimental spectra. The results will serve as introductory information for planning further experimental and in-depth theoretical studies on the new targeted anticancer therapies using cathinone and its derivatives as potential drugs and ordered carbon nanostructures as potential carriers.

## 2. Results and Discussion

### 2.1. Selection of Models and Theoretical Methodology

**Figure 1:**
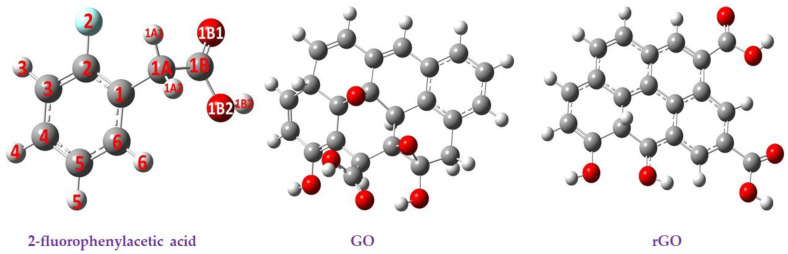
Structures of GO and rGO models prepared according to [35] and 2-fluorophenylacetic acid. Grey spheres represent carbon atoms, white ones hydrogen, red ones oxygen, and blue ones fluorine. The numbers on the atoms of 2-fluorophenylacetic acid represent the atom numbering used in the remainder of the article.

Flake graphene oxide was considered in this work as a potential drug carrier. This material has a complex structure of carbon sheets that tend to aggregate into stacks ranging from 3 to 10 layers [35,36,37]. Consequently, the modelling of such extensive systems using DFT methods would be very difficult. Therefore, simplified models were used in this work. The choice of flake size was based on the results of a previous study, where the interactions of graphene oxide and reduced graphene oxide (rGO) with selected porphyrins were studied using theoretical and experimental methods [35]. We further reduced the size of the GO and rGO models to more efficiently use the computational resources while maintaining the reliability of results. This simplification was based on our previous studies on the effect of GO model size on the strength of intermolecular H-bonds [38]. The analyzed models, together with the structures of o-fluorophenylacetic acid, are shown in Figure 1. As can be seen, these dimensions allow for the efficient placement of the entire F-derivative molecule either above the GO/rGO plane or in position, which allows for interactions with the functional groups only at the GO edges. To consider the influence of the GO functionalization degree on the type and strength of intermolecular interactions, both oxidized and reduced models were considered. In the case of the oxidized model (GO), polar substituents were placed both at the ends and on the surface of the graphene layer. To consider the effect of interactions with more or less polar GO surfaces, functional groups were placed only on one side of the plane. In the case of rGO, polar substituents were placed only at the edges, as reported previously [35]. In addition, finite models of ordered carbon nanostructures are also frequently used in the molecular modelling of the different chemical properties of nanomaterials [35,39,40]. Since the experimental NMR studies were measured in D_2_O, all calculations were performed in water using the PCM model. This solvent approximation does not allow for the calculation of the direct interactions of the studied molecules with solvent molecules. However, it allows one to consider GO/rGO–o-fluorophenylacetic acid interactions in a polar medium—the main goal of our study—with lower computational costs than in the explicit solvent model. Moreover, in previous studies using complexes with carbon nanotubes [33], we noted that using the PCM model could significantly complicate only the study of charge-assisted H-bonds. As will be demonstrated below, in this study, no complexes with the hopping hydrogen atom to the negatively charged oxygen atom were observed. Therefore, based on the obtained data, and also considering the introductory nature of our studies, discrete solvent molecules were not included in the molecular modelling. The direct solvent effect will be considered in further, more in-depth, theoretical and experimental studies of potential DDS cathinone–GO complexes. The pKa of the -COOH group of GO is approximately 6.6 and that of -OH is about 9 [41], while the pKa of -COOH of o-fluorophenylacetic acid is approximately 4 [42], and their protonated and deprotonated forms are considered in this work. To model the structural, and energetic properties of the GO–2-fluorophenylacetic acid complexes for which NMR data were obtained at a pH of about 2–3, GO and the F-derivative were considered in the protonated form. For complexes with a pH of about 7, both GO/rGO and F-derivative anions were considered.

Based on previous studies and the good performance of the B3LYP functional using empirical D3BJ Grimme’s correction for dispersion in predicting the structural and energetic parameters of complexes with non-covalent interactions, as well as small and medium-sized organic molecules [43,44,45], the optimization of the structures of GO, rGO, and their complexes was carried out using this methodology. Pople’s type 6-311++G** basis set was chosen due to the significant size of the studied molecular complexes, which limits the use of a more flexible and complete basis set and higher-level theory.

**Figure 2:**
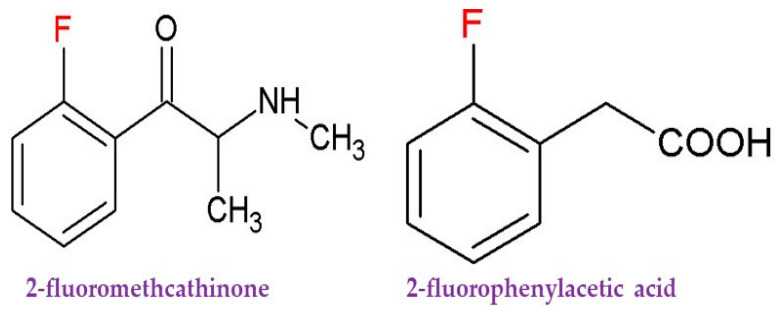
Comparison of the structures of 2-fluoromethcathinone and 2-fluorophenylacetic acid.

Since we did not have permission to conduct experimental studies using narcotics, we chose a non-psychotropic model of fluorinated cathinones—2-fluorophenylacetic acid. This choice was justified by the presence of a fluorinated benzene ring and a -CH_2_-COOH substituent in its molecular skeleton. This structure allows us to consider both the effect of the halogen and the curved aliphatic moiety on the formation of a complex with GO. Furthermore, the carboxyl group allows us to examine the influence of the substituent charge on the formation of non-covalent interactions with graphene oxide. As can be seen from Figure 2, 2-fluoromethcathinone, as an example of a fluorinated cathinone, also possesses a fluorinated benzene ring and an extensive N-substituent. A carbonyl group is present at the N-terminus, similar to the -COOH of fluorinated acid. Furthermore, the amine end will possess a positive charge at both acidic and a neutral pH [46]. Therefore, in the case of 2-fluorophenylacetic acid, the possibility of forming charge-assisted non-covalent interactions was also considered. Used cathinone model undoubtedly cannot be accepted as a full-fledged structural analogue of cathinone. However, the aim of this study was to examine the effects on the NMR spectrum and T_1_ relaxation times expected during the interaction of GO with a fluorinated aromatic structure with a spatial substituent and a charge. Furthermore, the structure of the expected GO–2-fluorophenylacetic acid complex was considered. The obtained results should form the basis for further predictions of the GO-fluorinated cathinone’s geometry, as well as simplifying the interpretation of experimental NMR data for these complexes.

### 2.2. Comparison of Theoretical ^1^H and ^13^C NMR Chemical Shifts, as Well as ^n^J_(C-F)_ Cuopling Constants with Experiment

^1^H and ^13^C chemical shifts, obtained from GIAO-calculated isotropic shieldings, as well as separately calculated spin–spin coupling constants ^n^J_(C-F)_, were obtained. For a better visualization, the theoretical and experimental chemical shift parameters in acidic and neutral media are summarized in Table 1. Based on the good performance in predicting ^1^H and ^13^C chemical shifts for fluorinated cathinones presented in our previous study [47], the OLYP functional was used to predict these NMR parameters for 2-fluorophenylacetic acid. The literature shows that structure optimization and ^1^H and ^13^C chemical shift predictions using different functionals do not significantly affect the quality of the spectral parameters [48]. Therefore, δ(^1^H) and δ(^13^C) were calculated for fluorinated acid optimized using the B3LYP functional. In our previous study [47], it was noted that the prediction of carbon-13 shifts reproduced the best experimental data for OLYP in the gas phase. For ^1^H, no direct dependence of the accuracy of the results on the polarity of the medium was observed. In the current study, the geometry of 2-fluorophenylacetic acid was optimized in water using the PCM model. The purpose of this chemical shift modelling was to simplify the assignment of experimental signals to specific acid atoms. Consequently, the relative positions of atoms in the spectrum were considered, and obtaining theoretical results with the highest possible accuracy was not mandatory. For this reason, δ(^1^H) and δ(^13^C) calculations were performed at the OLYP/6-311++G** level of theory in water (PCM model).

As can be seen from Table 1, the obtained theoretical values are close to the experimental data. These results formed the basis for further interpretation of the spectra of 2-fluorophenylacetic acid in its free form and in a complex with GO.

Similarly to chemical shifts, the selected experimental and theoretical ^n^J_(C-F)_ values in both media are presented in Table 2. Since our previous work focused only on δ(^1^H) and δ(^13^C), certain difficulties arose in choosing a calculation methodology. For this reason, based on the B3LYP/6-311++G** geometry, the C-F coupling constants were calculated using the B3LYP, OLYP, and B971 functionals. The choice of B971 was justified by the literature data on its good performance in predicting the SSCC for some biologically active compounds [49]. Calculations were performed in water using the PCM model, similarly to δ(^1^H) and δ(^13^C).

Based on the results presented in Table 2, it can be concluded that the results predicted by the B971 functional best reproduce the experimental data. Therefore, further interpretation of the C-F spin–spin coupling constants of 2-fluorophenylacetic acid in free form and in GO complexes was based on these data.

### 2.3. ^1^H, ^13^C Shifts and ^n^J_(C-F)_ Constants of 2-Fluorophenylacetic Acid–GO Complexes

During the experimental NMR studies, o-fluorophenylacetic acid and its complexes with GO were analyzed in acidic and neutral D_2_O solutions. The obtained ^1^H and ^13^C chemical shifts are presented in Table 3, while the selected SSCC constants are summarized in Table 4. To avoid data overload, the experimental spectra are presented in Figures S1–S8 in the SI. Each figure shows a separate ^1^H or ^13^C spectrum on the left panel and an enlargement of its aromatic region on the right panel. The measured chemical shifts were corrected for the position of 1,4-dioxane as a secondary reference (δ(^1^H) = 3.53 ppm and δ(^13^C) = 66.66 ppm with respect to TMS [50]).

From the ^1^H NMR spectra one cannot see any difference in the positions of individual peaks in free and complexed acid in both neutral and acidic solutions. Carbon-13 data are also the same for the free and complexed acid spectra. However, there is a very pronounced change in the carboxylic signal. It nearly completely disappeared in the presence of GO in a neutral environment. This effect is due to the formation of non-covalent H-bond with the -COOH groups of acid.

The shapes of ^1^H NMR signals were more informative. As can be seen in Figures S1 and S5, the addition of GO to an acidic solution of 2-fluorophenylacetic acid led to a significant broadening of the aromatic peaks in the ranges of approx. 7.3–7.4 and 7.1–7.2 ppm. This change in spectral pattern may indicate a tendency toward the formation of intermolecular interactions between graphene oxide and the studied F-derivative, in which the hydrogen atoms of the aromatic ring of the acid will play a key role. An even greater broadening of the signals can be seen in the case of GO-2-fluorophenylacetic acid complexes in a neutral medium compared to the acid solution (see Figures S3 and S7). Comparing the ^1^H NMR spectra of o-fluorophenylacetic acid complexes in acidic (Figure S5) and neutral (Figure S7) media, a strong tendency for spectral signals broadening in the range of approx. 7.3–7.4 ppm toward the neutral medium is observed. This may indicate both a greater tendency for GO-2-fluorophenylacetic acid complex formation in the neutral medium and the more significant role of acid H4 and H6 atoms in association with graphene oxide fragments compared to H3 and H5 atoms.

There are very small changes in C-F coupling constants (the biggest difference is about 0.3 Hz for ^1^J_C-F_ between free acid and its complex with GO in acidic solution). Therefore, looking only at the spectral pattern change (disappearance of -COOH signal in the ^13^C spectrum and broadening of selected ^1^H NMR signals) we could conclude that a non-covalent GO–acid complex is formed.

**Figure 3:**
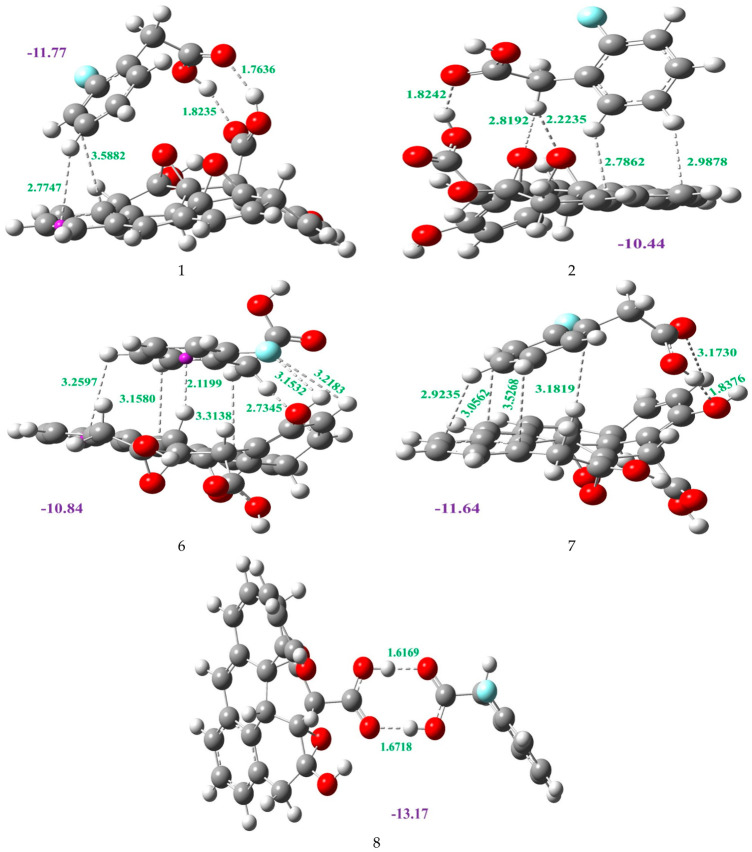
Structures of the most stable complexes of protonated GO models with 2-fluorophenylacetic acid (simplified acidic environment) modelled in water using the PCM model. The lengths of non-covalent intermolecular interactions (in Å) are shown in green, and the BSSE-corrected total binding energy (in kcal/mol) is shown in purple. Carbon atoms are shown as grey spheres, oxygen atoms as red spheres, fluorine atoms as blue spheres, and hydrogen atoms as white spheres.

For a better explanation, in which intermolecular interactions in GO-o-2-fluorophenylacetic acid solutions play a decisive role in stabilizing the complexes and potentially lead to changes in the ^1^H and ^13^C NMR spectral parameters, the results of in silico studies are presented below. To avoid data overload in the article, Figure 3 shows only the most stable complexes with the GO model at an acidic pH (protonated forms). The structural parameters for all complexes, obtained by modelling the non-covalent interactions of GO models with o-fluorophenylacetic acid in simplified acidic and neutral media, are presented in the figures in Table S1 in the SI.

**Figure 4:**
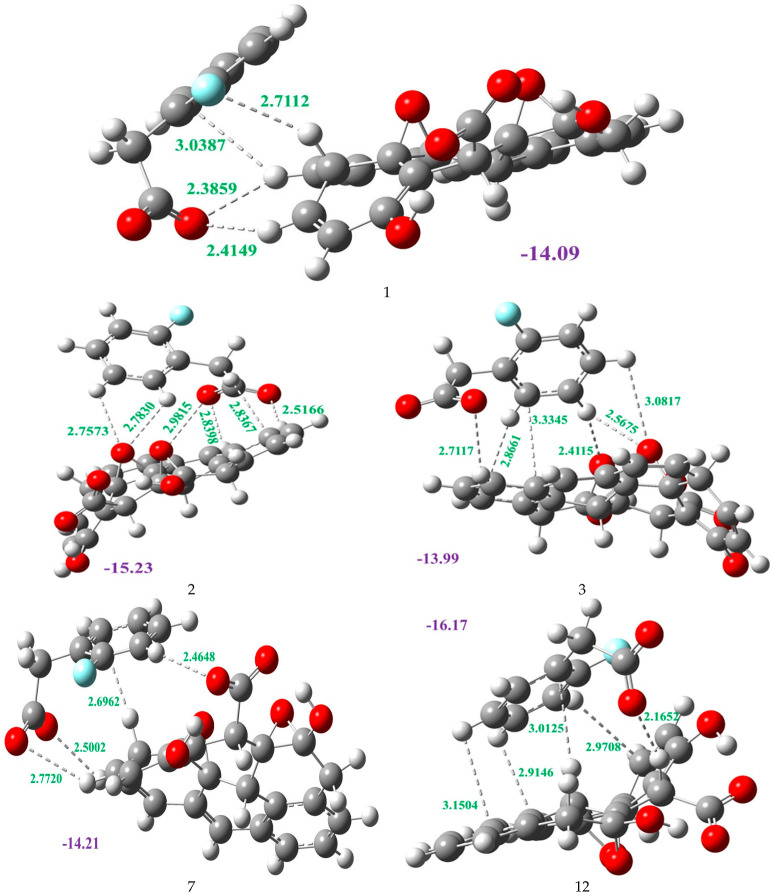
Structures of the most stable complexes of deprotonated GO models with 2-fluorophenylacetic acid (simplified neutral environment) modelled in water using PCM model. The lengths of non-covalent intermolecular interactions (in Å) are shown in green, and the BSSE-corrected total binding energy (in kcal/mol) is shown in purple. Carbon atoms are shown as grey spheres, oxygen atoms as red spheres, fluorine atoms as blue spheres, and hydrogen atoms as white spheres.

Analyzing all the complexes in Figure 3, a general trend toward the formation of H-bonds involving the -COOH group of the F-derivative is observed. Moreover, as seen in the case of complex 8, the formation of a classical non-covalent dimer between two carboxyl groups of the GO model and 2-fluorophenylacetic acid leads to the complex with the strongest interactions (E_BSSE_ = 13.17 kcal/mol). Furthermore, when the F-derivative is located above the GO plane, the formation of the shortest H-bonds involving -COOH is hindered, and the intermolecular interactions of the H-π and π-π types act as additional stabilizing factors for the complexes. For example, in complex 1, it could be noted that in addition to the H-bonds involving acid -COOH, which are longer than in complex 8, the H4 atom participates in the H-π (2.7747 Å) bond, and the C3 atom in the π-π stacked interaction (3.5882 Å). Consequently, complex 1 has an E_BSSE_ value close to that of complex 8. Other important intermolecular interactions stabilizing the complexes are the H-bonds of F-derivative with the less polar than -COOH, -OH and epoxy groups of GO. As could be seen for complexes 2, 6, and 7, the arrangement of acid -COOH relative to -COOH from GO, which hinders or makes impossible the formation of two strong H-bonds, is compensated by the large number of interactions among H-π, π-π types and less polar H-bridges. Therefore, the E_BSSE_ values of these complexes are only about 2–3 kcal/mol lower than in case of the most stable complex 8. It is also worth noting that all the most stable GO-2-fluorophenylacetic acid complexes (except complex 8) modelled in a simplified acidic medium involve the H4, H6, C4, and C6 atoms. It is also worth noting that the fluorine atom in the *ortho* position was positioned relative to GO such that it did not participate in non-covalent interactions with its surface (see Figure 4). Consequently, an agreement between the theoretical structural-energy results and the experimental ^1^H and ^13^C NMR parameters can be observed. Based on the obtained data, it could be assumed that in the case of GO fragments with a high degree of polar groups’ functionalization, complexes with 2-fluorophenylacetic acid, stabilized by interactions with -COOH substituents at the GO edges, will be some of the most privileged in an acidic medium. Another possibility is the location of the 2F-derivative above the surface of the GO layer, which will lead to a weakening of the H-bonds involving -COOH but stabilize the complex through a large number of weak H-π, π-π interactions and H-bonds with a less polar -OH and epoxy groups of GO. In a number of weaker intermolecular interactions, those involving the H4, H6, C4, and C6 atoms of the acid will predominate. It is also worth noting that the fluorine atom apparently does not form intermolecular H-bonds.

Similar to GO-2-fluorophenylacetic acid in a simplified acidic medium (protonation of GO and acid), GO–acid complexes were modelled in a neutral medium (water in the PCM model using the anionic forms of both compounds). The structures of the most stable complexes in this series are shown in Figure 4.

As can be seen from Figure 4, the participation of the -COO^-^ groups of the F-derivative and GO plays a key role in stabilizing the complexes in a neutral medium. As can be seen for the most stable complexes, 1, 2, and 12, the charged H-bonds are longer compared to the H-bridges involving -COOH; however, the E_BSSE_ values are higher or close to the E_BSSE_ of complex 8, which is most stable in an acidic medium (see Figure 3). In addition, it is worth noting the tendency, similar to an acidic medium, of the additional stabilization of the complex by weaker H-π, π-π, and the fewer polar H-bonds in cases where the location of the 2F-derivative does not allow for the formation of the shortest and strongest H-bonds with GO. It is also worth noting the trend, similar to an acidic medium, of the participation of H4, H6, C4, and C8 acid atoms in intermolecular interactions with GO. Furthermore, it was observed that the fluorine atom participates in a low-energy H-bond (2.7112 Å) with GO in one of the most stable complexes 1 (see Figure 4). Consequently, it is also worth noting the consistency of the experimental data for GO-2-fluorophenylacetic acid complexes compared with the theoretical ones in a neutral medium. It could be assumed that in an experimental solution with a neutral pH, 2-fluorophenylacetic acid tends to be located above the most polar fragments of GO, so that both the strongest charge-conjugated H-bonds and the greatest possible number of weak non-covalent interactions (H-π, π-π, and low-polarity H-bridges) are formed simultaneously.

**Figure 5:**
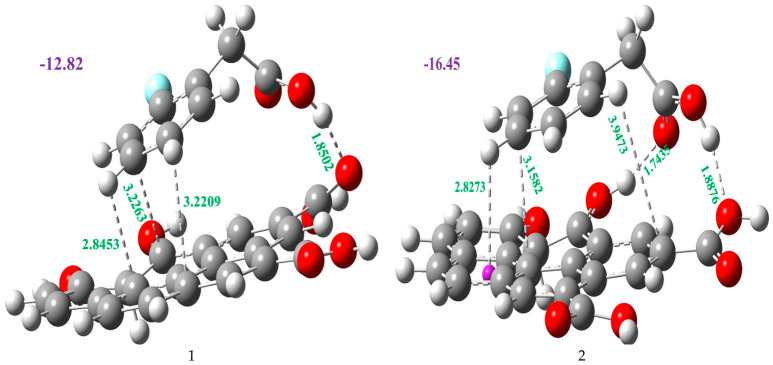

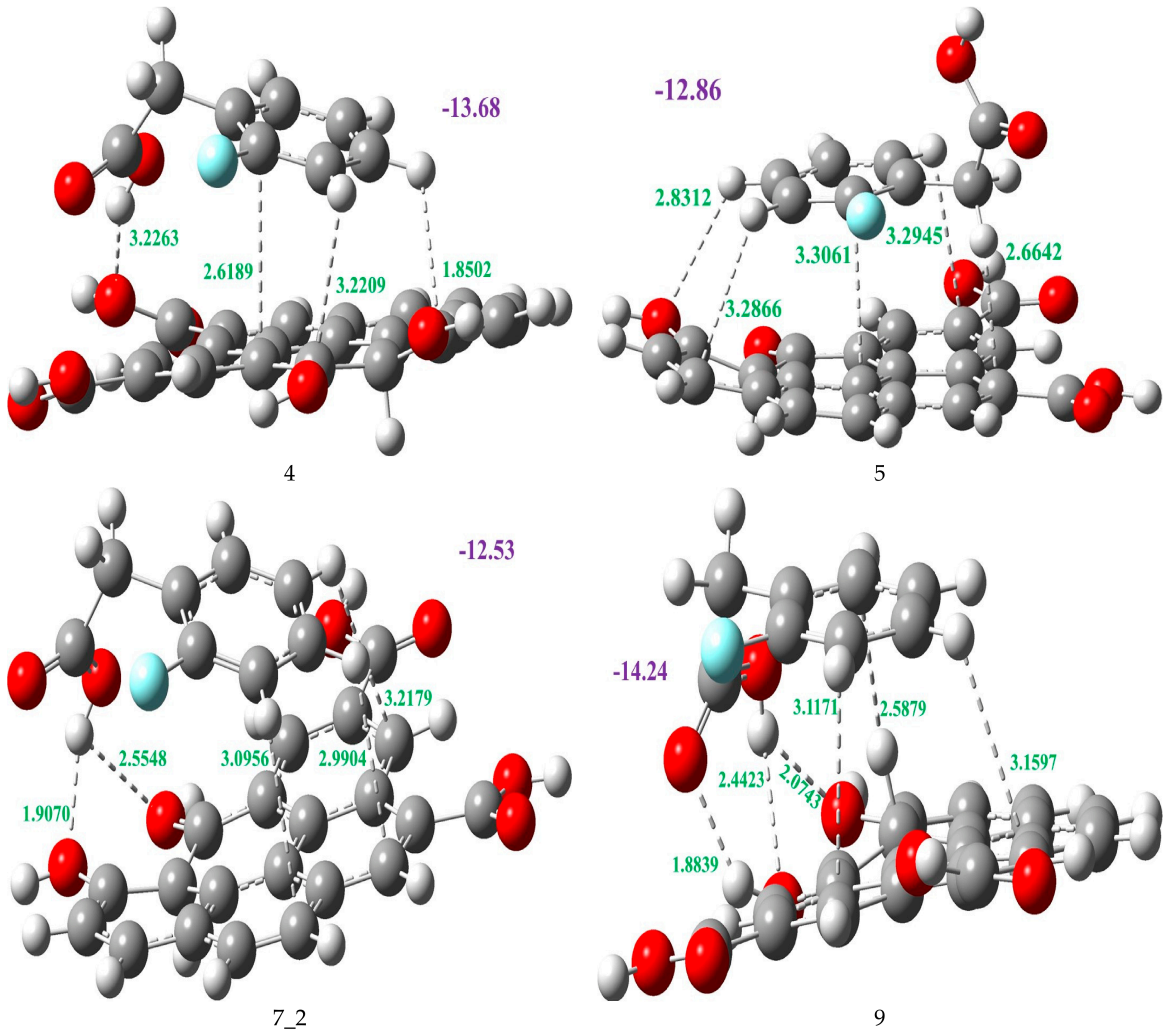
Structures of the most stable complexes of protonated rGO models with 2-fluorophenylacetic acid (simplified acidic environment), modelled in water using a PCM model. The lengths of the non-covalent intermolecular interactions (in Å) are shown in green, and the BSSE-corrected total binding energy (in kcal/mol) is shown in purple. Carbon atoms are shown as grey spheres, oxygen atoms as red spheres, fluorine atoms as blue spheres, and hydrogen atoms as white spheres.

**Figure 6:**
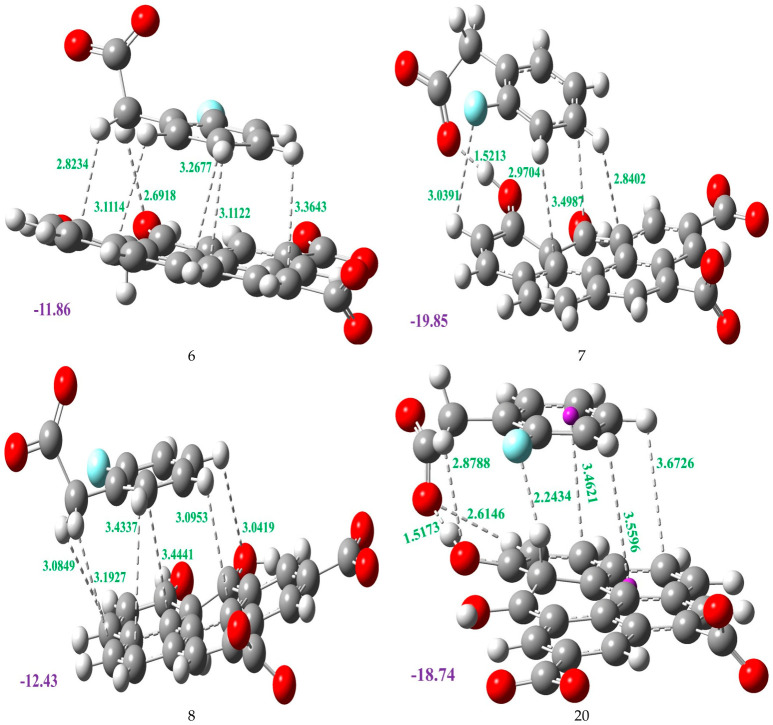
Structures of the most stable complexes of deprotonated rGO models with 2-fluorophenylacetic acid (simplified neutral environment), modelled in water using a PCM model. The lengths of non-covalent intermolecular interactions (in Å) are shown in green, and the BSSE-corrected total binding energy (in kcal/mol) is shown in purple. Carbon atoms are shown as grey spheres, oxygen atoms as red spheres, fluorine atoms as blue spheres, and hydrogen atoms as white spheres.

Since graphene oxide does not have a homogenous structure [35], complexes with its partially reduced models (rGO) were also modelled in both simplified acidic (protonated structures) and neutral (deprotonated structures) media. Similar to the GO results, Figure 5 shows the most stable rGO-2-fluorophenylacetic acid complexes in an acidic medium, while Figure 6 shows those in a neutral medium.

In both neutral and acidic environments, a decisive role of H-bonds involving the carboxyl group of the 2F-derivative was observed in complexes with rGO, similar to that observed for complexes with GO. It is worth noting that in the case of reduced graphene oxide, where polar functional groups predominate at the edges [35], the aromatic nature of the carbon skeleton forces the acid to be positioned so as to facilitate both strong H-bonds involving -COOH and a large number of low-polar H-π and π-π intermolecular interactions. Consequently, for rGO, an even greater influence of intermolecular H-π and π-π interactions involving H4, H6, C4, and C6 acid atoms on complex stabilization could be observed as compared to GO complexes. An apparent lack of fluorine atom intermolecular H-bond formation can also be observed in rGO complexes in both acidic and neutral environments.

Summarizing all the theoretical results, it could be suspected that in real acidic and neutral aqueous solutions, two categories of non-covalent intermolecular interactions could play a decisive role in graphene oxide–2-fluorophenylacetic acid complexes’ stabilization. There are strong H-bonds involving the -COOH/-COO^-^ groups of 2-fluorophenylacetic acid and GO, as well as weak H-π, π-π, and low-polarity H-bridges. The leading roles in the formation of low-energy intermolecular interactions will be played by the H4, H6, C4, and C6 atoms in the acid. Entire acid molecules positioned above the surface of the GO fragment will be the most privileged in the case of both more- and less-functionalized GO fragments, since this position allows for the formation of the maximum number of the aforementioned types of intermolecular molecular bonds and maximizes the stabilization of the complex.

### 2.4. Relaxation Times T_1_

In addition to classical ^1^H and ^13^C NMR measurements, the T_1_ relaxation times were also measured for aqueous solutions of free o-fluorophenlylacetic acid and its complexes with GO in acidic and neutral media. In this case, the mobility of water molecules and complex components in different pHs was studied. The decrease in relaxation time T_1_ is related to a decrease in the mobility of the studied acid molecules in the presence of GO plates. The obtained T_1_ values for the water peak and selected aromatic and aliphatic protons of 2-fluorophenlylacetic acid are presented in Table 5. It is evident that the presence of GO significantly shortened the T_1_ value of water (from 1.14 s to 0.41 s in acidic medium and from 1.1853 s to 1.697 s in a neutral environment). On the other hand, the presence of GO has a marked impact on the dynamics of H4 and H5 aromatic protons. For example, there is a pronounced decrease in T_1_ of H4 in an acidic environment upon the addition of GO (T_1_ decreases from 6.152 s to 1.904 s). All these changes indicate the slower dynamics of the potentially formed GO-2–fluorophenylacetic acid complex with respect to the free acid solution. They also indicate the higher stability of the complex formed in the neutral environment.

The presence of paramagnetic Mn^2+^ ions in GO was confirmed by Panich et al. [51]. For this reason, we decided to check the impact of Mn^2+^ cations on linewidth and the T_1_ relaxation time of water and CH_2_ signals. Table 6 shows the ^1^H NMR linewidths (in Hz) and relaxation times T_1_ (in s) of the residual HOD signal in D_2_O and CH_2_ fragments from acid in the studied systems. These two parameters are related to dynamic processes at the molecular level and are sensitive to the composition of the solution.

In neat, heavy water, a narrow HOD signal of 0.40 Hz linewidth is shown, with a relatively long relaxation time of 4.370 s. In the presence of EDTA this grows somehow broader (0.60 Hz) and is characterized by a significantly longer T_1_ longitudinal (or spin-lattice) relaxation time of 15.560 s. The presence of acid in water introduces more significant changes. Thus, both HOD and CH_2_ signals’ linewidths are about 3.39 Hz and 3.50 Hz and the corresponding T_1_ values are about 1.140 and 2.590 s. The presence of very small amount of GO in water significantly broadens the HOD signal (13.3 Hz) and shortens its relaxation time (T_1_ of 0.76 s). At this point, it is worth noting that the Hummers method [52] of producing GO is based on graphite oxidation with a mixture of KMnO_4_ and H_2_SO_4_. The presence of paramagnetic manganese ions in GO cannot easily be fully washed out with water. According to detailed EPR and NMR studies by Panich et al. [51], in GO are present both some paramagnetic defects and Mn^2+^ ions. The presence of unpaired electrons results in a decrease in the T_2_ spin–spin (or transverse) relaxation time of water, as well as shortening its measured T_1_ relaxation time. Therefore, one could expect line-broadening in a water solution of GO (or its suspension). However, these effects work at very short distances and decrease with 1/R^6^ [53,54], where R is the distance from a paramagnetic centre to the individual hydrogen or carbon atom. In the next step, we measured a mixture of GO and acid and noticed further a increase in HOD and CH_2_ signals’ linewidth (21 and 7 Hz), accompanied by significant T_1_-shortening (below 1 s). On the other hand, a mixture of acid and 10 mM EDTA resulted in very narrow HOD and CH_2_ signals (0.8 and 1.6 Hz). Since GO contains paramagnetic manganese ions, we decided to study the effect of Mn^2+^ on water and CH_2_ signals. The following concentrations of MnCl_2_ in water were prepared: 10^−6^, 10^−5^ and 10^−4^ mM. As result of water’s interaction with a paramagnetic centre, the water linewidths were 4.9, 5.8 and 10.3 Hz, respectively. The T_1_ value for the HOD signal in the latter case was fairly short (0.8 s). Thus, in subsequent studies, we used the 10^−4^ mM manganese ion solution.

As expected, the addition of a paramagnetic manganese solution to acid resulted in both the significant broadening of both signals (linewidths of 9 and 7 Hz) and a decrease in T_1_ to 1.4 and 1.8 s, respectively. This result suggests a fast exchange of water molecules from the first hydration sphere of free Mn^2+^ ions with bulk water, as well as with the hydration sphere of Mn–acid complexes. The latter effect is weaker due to the larger distance between the paramagnetic ion and CH_2_ protons (about 4 Å). However, the addition of an EDTA complexing agent to the Mn^2+^ solution results in a very narrow HOD signal (0.6 Hz). This indicates the very efficient removal of free manganese cation and, as result of complexation, its insulation from bulk water. Interestingly, EDTA is also able to complex manganese cation originating from GO (the resulting water linewidth is 1.3 Hz and T_1_ is relatively long, 11.9 s). It is important to note that the addition of excess of manganese ions to GO with EDTA produces an HOD linewidth of 0.8 Hz. This shows that EDTA is still able to efficiently bind the paramagnetic ions in this solution. In the last experiment, the manganese solution and EDTA were added to a mixture of GO and acid. In this case, fairly narrow HOD and CH_2_ signals were observed (1.4 and 2.9 Hz). The corresponding T_1_ values were about 1.9 and 1.4 s, respectively.

The results above support the presence of paramagnetic centres/ions in GO and support the results reported by Panich et al. [51]. The most interesting composition in our studies was GO + acid, showing significant broadening of HOD and CH_2_ signals (21 and 5.6 Hz, which is larger than that for Mn^2+^ released from GO). Therefore, an additional mechanism is postulated in this case: an immobilization of acid on GO edges by the formation of strong H-bonds with the COOH group.

## 3. Future Perspectives

**Figure 7:**
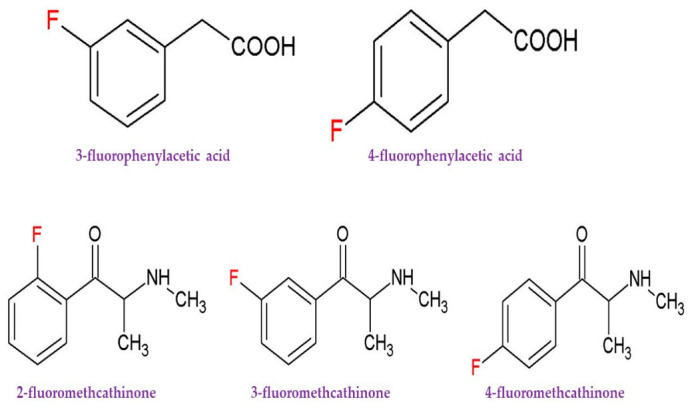
Structures of the 3- and 4-fluorophenylacetic acid and 2-, 3- and 4-fluoromethcathinone (according to [3]).

In this study, we obtained experimental and theoretical data for one F-derivative of phenylacetic acid. Further experimental NMR and in silico studies for m- and p-fluorophenylacetic acids (see Figure 7) are planned. It could be analyzed how the position of the fluorine atom relative to -COOH affects the nature and strength of interactions with GO in acidic and neutral media. We also plan to study the effect of a gradient change in pH values from acidic through slightly acidic and neutral to basic to analyze the effect of pH on the spectral data and the underlying structural and energetic modifications in GO–acid complexes. Figure 7 also shows the structure of cathinone and its selected fluorinated derivatives. As can be seen, cathinones have an amine terminus, with a pKa of 8–10 [46]. Therefore, based on our previous theoretical studies [33] and the current results, it could be assumed that the cationic form of cathinones, which predominates at neutral and acidic pHs, may lead to an even greater number of charge-assisted H-bridges with GO. Consequently, similar to the F-derivatives of phenylacetic acid, greater stabilization of the complex in a neutral medium could be expected. However, this requires confirmation and will be tested, along with the effect of decreasing pH, in subsequent studies.

## 4. Materials and Methods

### 4.1. Materials

2-fluorophenylacetic acid, D_2_O and MnCI_2_ were purchased from Sigma-Aldrich (Merck Group, Saint Luis, MO, USA). Graphene oxide samples were obtained from Artur Małolepszy lab (Warsaw University of Technology, Warsaw, Poland). The production and purification details are reported in [55].

### 4.2. NMR Measurements

^1^H and ^13^C NMR spectra of all acids, graphene oxide and GO–acid complexes were measured in D_2_O at room temperature using BRUKER ULTRASHIELD™ PLUS AV2 spectrometer (600.22 MHz for protons, Bruker Corporation, Billerica, Massachusetts, USA). Proton and carbon-13 spectra were determined with reference to an internal standard (1,4-dioxane). In case of the ^1^H spectra, the following parameters were used: 64 scans, 6 s acquisition time and 0.5 s relaxation delay. In case of the ^13^C spectra, the following parameters were used: 256 scans. T_1_ experiments [56] were performed for the proton signals of water, studied acids and GO–acid complexes. The inversion and recovery (IR) method was used with the longest delay of 20 s.

### 4.3. Theoretical Methods

All calculations were performed using Gaussian 16 C. 02 software [57]. Density functional theory (DFT) was used for the prediction of structural and energetic parameters of all studied free molecules and complexes. All structures were optimized at the B3LYPD3BJ/6-311++G** level of the theory with the tight convergence criterion [58,59,60,61,62]. Isotropic chemical shieldings were calculated using the GIAO method [63,64] on the OLYP/6-311++G** [65] level of the theory. TMS calculated at the same level of theory as acid was used as a theoretical reference for both ^1^H and ^13^C chemical shifts. Indirect spin–spin coupling constants were calculated on B3LYP/6-311++G**, OLYP/6-311++G** and B971/6-311++G** [66] levels of theory using the mixed-basis set approach for efficient calculations. All calculations were performed in water using the PCM solvation model [67]. For simplified analyses of pH’s effect on the structural and energetic parameters of studied free compounds and complexes, systems with protonated (acidic environment model) and deprotonated (neutral environment model) molecules were predicted. IR spectroscopic parameters were calculated for all optimized structures. The luck of imaginary frequencies was used as a criterion for the obtained equilibrium structure. The binding energy ∆E_GO−Acid_ of complexes (kcal/mol) was determined using Formula (1):

ΔE_GO-Acid_ = (E_GO-Acid_ – E_GO_ – E_Acid_) ∗ 627.509 (1)

where E is the electronic energy of the studied complex or its fragments. The obtained values were corrected for the basis set superposition error (BSSE) using the counterpoise method [68].

## 5. Conclusions

Based on the experimental and theoretical data, it was observed that 2-fluorophenylacetic acid has a stronger tendency to aggregate with GO in a neutral medium compared to in acidic environments. This is evident both from the broadening of selected peaks in the ^1^H NMR spectra and from the disappearance of the carbon peak from the carboxylic acid. It is suggested that interactions between the carboxyl group of the acid and GO play a crucial role in stabilizing the complexes. Furthermore, the H4, H6, C4, and C6 atoms also play a leading role in forming weaker non-covalent intermolecular interactions, a large number of which can synergistically stabilize the complex in both acidic and neutral media. The results of the molecular modelling are consistent with the experimental data. Based on in silico data, it is suggested that the most beneficial arrangement of 2-fluorophenylacetic acid is when the entire molecule lies over the surface of GO. This geometry of the complex allows for the formation of both charge-assisted (neutral environment) and classical H-bonds (acidic environment) with the participation of the carboxyl groups of GO and F-derivative, as well as a number of other interactions involving the carbon and hydrogen atoms of the aromatic ring of the acid. In particular, the significant shortening of water and selected acid proton signals’ relaxation time T_1_ (especially in the neutral environment) proved the formation of an acid–GO associate. An immobilization of acid on GO edges through the formation of strong H-bonds with the -COOH group is postulated and a possible interference effect of Mn^2+^ ions on complex formation is excluded. These results are promising for future studies of GO–cathinone complexes.

## Figures and Tables

**Table 1:** Comparison of calculated and experimental δ(^1^H) and δ(^13^C) values (in ppm) of o-fluorophenylacetic acid in acidic and neutral environments.

Atoms ^1^	δ_calc_		δ_exp_	
	R-COOH	R-COO^-^	R-COOH	R-COO^-^
H1A1 and H1A2	3.9343	3.7771	3.7819	3.3365
H3	7.3763	7.2632	7.1646	6.8995
H4	7.7002	7.4740	7.3286	7.0619
H5	7.5058	7.3685	7.1990	6.9408
H6	7.6762	7.4507	7.3712	7.0827
C1	123.7539	129.7945	120.3137	124.4560
C1A	40.0636	42.6533	33.2603	37.7716
C1B	172.3316	171.1864	175.0743	180.0295
C2	165.5005	165.7784	159.9316	161.1200
C3	114.1620	113.4599	114.2672	115.1997
C4	128.7500	125.4284	128.5658	128.6276
C5	123.3475	122.3064	123.5014	124.3663
C6	132.3162	131.4907	130.7496	131.7464

^1^ The numbering of atoms is presented according to Figure 1.

**Table 2:** Comparison of calculated and experimental ^n^J_(C-F)_ values (in Hz) of o-fluorophenylacetic acid in acidic and neutral environments.

Couplings ^1^	Calc.		Exp.	
	R-COOH	R-COO^-^	R-COOH	R-COO^-^
**B3LYP**				
^1^J_(C2-F2)_	303.8150	300.1750	243.7509	243.1321
^2^J_(C1-F2)_	14.3532	14.6832	16.4654	16.2088
^2^J_(C3-F2)_	19.3546	20.8353	21.2495	21.7023
^3^J_(C6-F2)_	2.9032	4.3578	4.1956	4.6634
^3^J_(C4-F2)_	6.7001	6.5395	8.3006	8.1950
^4^J_(C5-F2)_	4.5171	4.3697	3.5315	3.4863
**OLYP**				
^1^J_(C2-F2)_	335.1980	330.1580	243.7509	243.1321
^2^J_(C1-F2)_	7.2340	8.2675	16.4654	16.2088
^2^J_(C3-F2)_	12.9479	14.3234	21.2495	21.7023
^3^J_(C6-F2)_	3.1632	4.8537	4.1956	4.6634
^3^J_(C4-F2)_	6.6797	6.3673	8.3006	8.1950
^4^J_(C5-F2)_	3.0244	2.8641	3.5315	3.4863
**B971**				
^1^J_(C2-F2)_	295.9310	292.3940	243.7509	243.1321
^2^J_(C1-F2)_	15.5674	15.9000	16.4654	16.2088
^2^J_(C3-F2)_	20.1418	21.6329	21.2495	21.7023
^3^J_(C6-F2)_	3.6752	5.1590	4.1956	4.6634
^3^J_(C4-F2)_	7.5058	7.3383	8.3006	8.1950
^4^J_(C5-F2)_	4.3501	4.1985	3.5315	3.4863

^1^ The numbering of the atoms is presented according to Figure 1.

**Table 3:** Experimental δ(^1^H) and δ(^13^C) values (in ppm) of o-fluorophenylacetic acid and its complexes with GO ^1^.

Atoms ^2^	δ_acid_		δ_complex_	
	R-COOH	R-COO^-^	R-COOH	R-COO^-^
H1A1 and H1A2	3.7824 (s)	3.3365 (s)	3.5575 (s)	3.3395 (s)
H3	7.1646 (m)	6.8995 (m)	6.9448 (m)	6.9000 (m)
H4	7.3286 (td)	7.0619 (m)	7.1069 (m)	7.0697 (m)
H5	7.1990 (td)	6.9408 (td)	6.9785 (m)	6.9404 (m)
H6	7.3712 (m)	7.0827 (m)	7.1500 (m)	7.0697 (m)
C1	120.3137 (d)	124.4560 (d)	121.4845 (d)	124.4593 (d)
C1A	33.2603 (s)	37.7716 (d)	34.4462 (s)	37.8007 (s)
C1B	175.0743 (s)	180.0295 (s)	176.2473 (s)	-
C2	159.9316 (d)	161.1200 (d)	161.0558 (d)	161.1194 (d)
C3	114.2672 (d)	115.1997 (d)	115.3828 (d)	115.1996 (d)
C4	128.5658 (d)	128.6276 (d)	129.6675 (d)	128.6291 (d)
C5	123.5014 (d)	124.3663 (d)	124.6187 (d)	124.3658 (d)
C6	130.7496 (d)	131.7464 (d)	131.8674 (d)	131.7461 (d)

^1^ Multiplicity in parenthesis. Some ^13^C signals are split by coupling with fluorine; ^2^ the numbering of atoms is presented according to Figure 1.

**Table 4:** Selected experimental ^n^J_(C-F)_ (in Hz) values of o-fluorophenylacetic acid and its complexes with GO in neutral and acidic environments.

Couplings ^1^	Acid		Complex	
	R-COOH	R-COO^-^	R-COOH	R-COO^-^
^1^J_(C2-F2)_	243.7509	243.1321	244.0527	243.1472
^2^J_(C1-F2)_	16.4654	16.2088	16.1937	16.2843
^2^J_(C3-F2)_	21.2495	21.7023	21.4457	21.7325
^3^J_(C6-F2)_	4.1956	4.6634	4.1503	4.6332
^3^J_(C4-F2)_	8.3006	8.1950	8.2553	8.1799
^4^J_(C5-F2)_	3.5315	3.4863	3.4561	3.4561

^1^ The numbering of atoms is presented according to Figure 1.

**Table 5:** T_1_ relaxation times (in s) of selected peaks in o-fluorophenylacetic acid in free form and in complexes with GO in neutral and acidic environments.

Atoms ^1^	Acid		Complex	
	R-COOH	R-COO^-^	R-COOH	R-COO^-^
H_2_O	1.140	1.853	0.410	1.697
H4	6.152	6.113	1.904	-
H5	4.797	7.443	1.530	-
H1A	2.115	2.571	0.960	0.630

^1^ The atom numbering is presented according to Figure 1.

**Table 6:** Linewidth and T_1_ relaxation times in the studied solutions in D_2_O.

Solution	*v*_1/2_, Hz		T_1_, s	
	HOD	CH_2_ (Acid)	HOD	CH_2_ (Acid)
Neat D_2_O	0.40	-	4.370	-
EDTA	0.60	-	15.560	-
Acid	3.39	3.50	1.140	2.590
GO	13.3	-	0.756	-
GO + Acid	21	5.60	0.392	0.906
Acid + 10mM EDTA	0.83	1.62	8.873	0.740
10^−4^ Mn^2+^	10.3	-	0.842	-
Mn^2+^ + Acid	9.2	7.00	1.399	1.754
Mn^2+^ + 100 mM EDTA	0.63	-	3.918	-
GO + 100 mM EDTA	1.31	-	11.870	-
GO + 10 mM EDTA	4.25	-	1.243	-
GO + Mn^2+^ + 10 mM EDTA	0.84	-	3.080	-
GO + Acid + Mn^2+^ + 10 mM EDTA	1.42	2.88	1.867	1.395

## Data Availability

Data are contained within the article and Supplementary Materials.

## Supplementary Materials

The following supporting information can be downloaded at: https://www.mdpi.com/article/10.3390/molecules31111801/s1, Figure S1: ^1^H NMR spectrum of 2-fluorophenylacetic acid in acidic environment; Figure S2: ^13^C NMR spectrum of 2-fluorophenylacetic acid in acidic environment; Figure S3: ^1^H NMR spectrum of 2-fluorophenylacetic acid in neutral environment; Figure S4: ^13^C NMR spectrum of 2-fluorophenylacetic acid in neutral environment; Figure S5: ^1^H NMR spectrum of 2-fluorophenylacetic acid in acidic environment after GO addition; Figure S6: ^13^C NMR spectrum of 2-fluorophenylacetic acid in acidic environment after GO addition; Figure S7: ^1^H NMR spectrum of 2-fluorophenylacetic acid in neutral environment after GO addition; Figure S8: ^13^C NMR spectrum of 2-fluorophenylacetic acid in neutral environment after GO addition; Table S1: Structures of 2-fluorophenylacetic acid complexes with graphene oxide (GO) and reduced graphene oxide (rGO) models optimized at the B3LYPD3BJ/6-311++G** level of theory in water using the PCM model. The lengths of non-covalent intermolecular interactions (in Å) are shown in green, and the BSSE-corrected total binding energy (in kcal/mol) is shown in purple. Carbon atoms are shown as grey spheres, oxygen atoms as red spheres, fluorine atoms as blue spheres, and hydrogen atoms as white spheres.
