# Supplementary material for: Experimental 1H, 13C and T1 NMR Studies of Graphene Oxide Interactions with 2-Fluorophenylacetic Acid as a Fluorinated Cathinone Model Supported by Molecular Modelling
Source: Molecules. 2026 May 24;31(11):1801. doi: 10.3390/molecules31111801 (PMC13258710; doi:10.3390/molecules31111801)
Supplement: Supplementary file 1 [file molecules-31-01801-s001.zip › molecules-4301797-supplementary.pdf]

SUPPORTING INFORMATION

for

Experimental ^{1}H, ^{13}C and T_1 NMR studies of graphene oxide interactions with 2-fluorophenylacetic acid as a fluorinated cathinone model supported with molecular modelling

by

Natalina Makieieva*, Michał Jewgiński, Artur Małolepszy and Teobald Kupka*

Table S1. Structures of 2-fluorophenylacetic acid complexes with graphene oxide (GO) and reduced graphene oxide (rGO) models optimized at the B3LYPD3BJ/6-311++G** level of theory in water using the PCM model. The lengths of non-covalent intermolecular interactions (in Å) are shown in green, and the BSSE-corrected total binding energy (in kcal/mol) is shown in purple. Carbon atoms are shown as grey spheres, oxygen atoms as red spheres, fluorine atoms as blue spheres, and hydrogen atoms as white spheres.

**GO – acidic pH**	
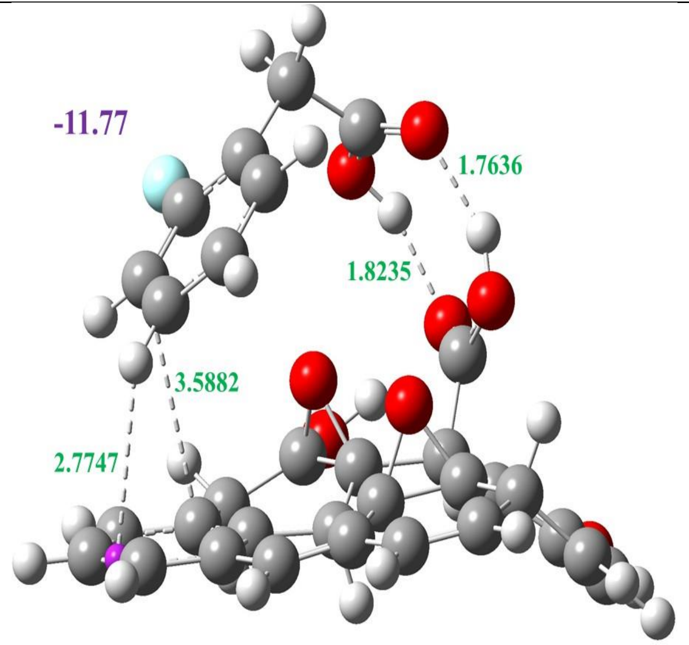	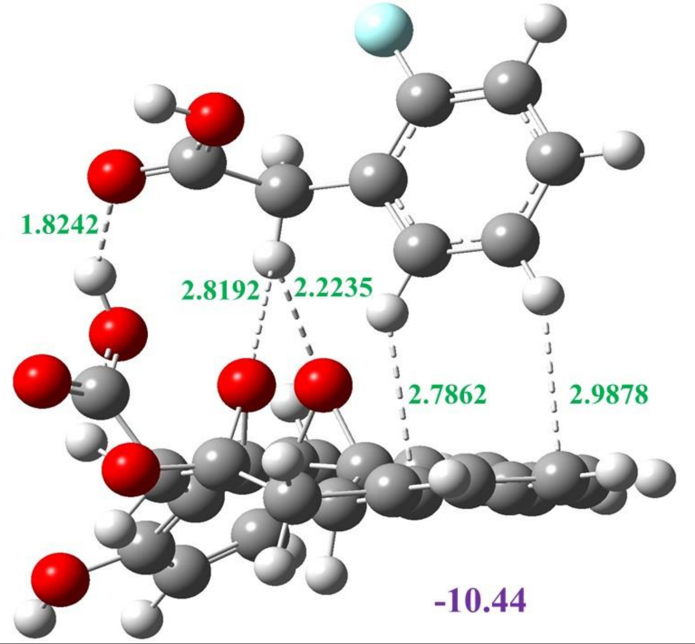
1	2
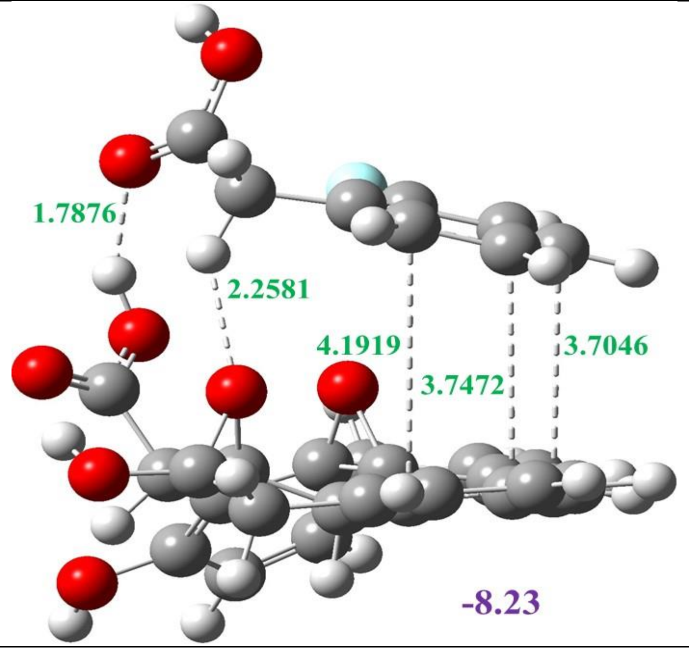	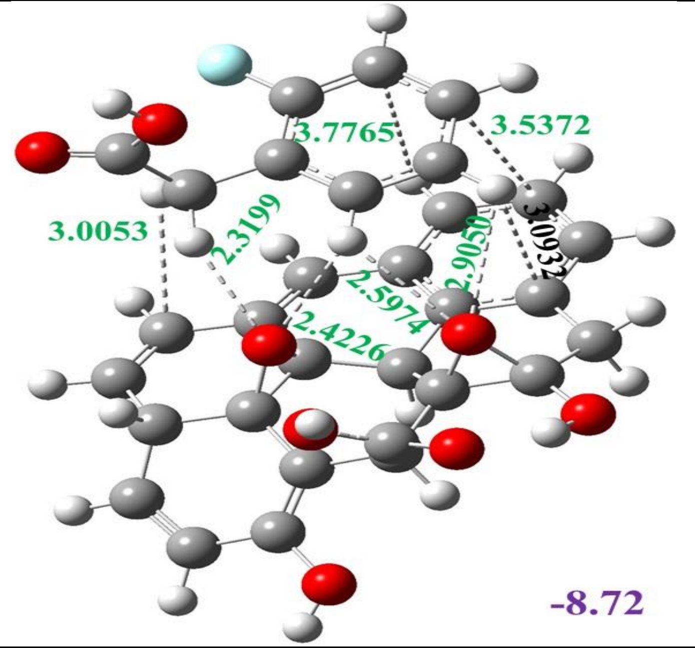
3	4

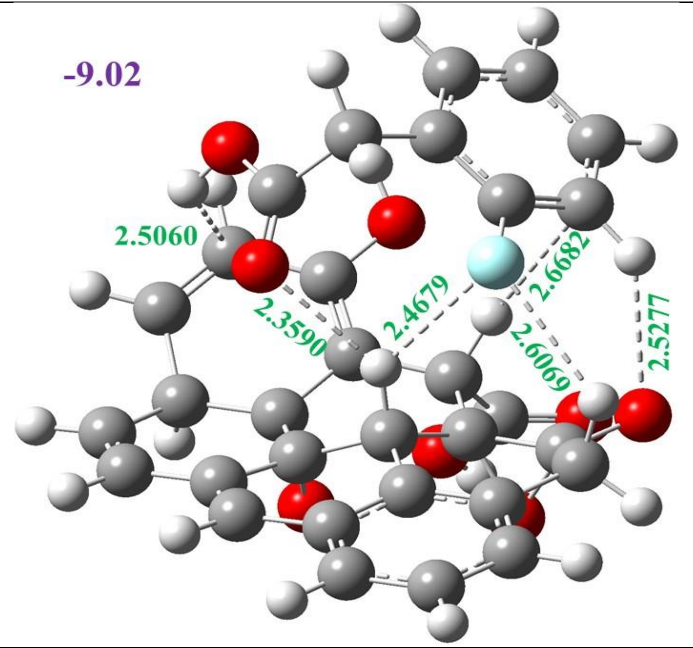

5

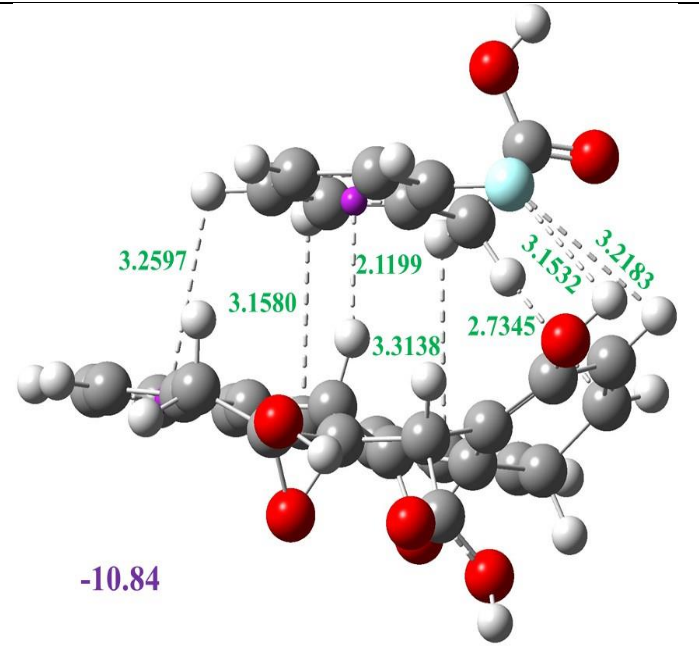

6

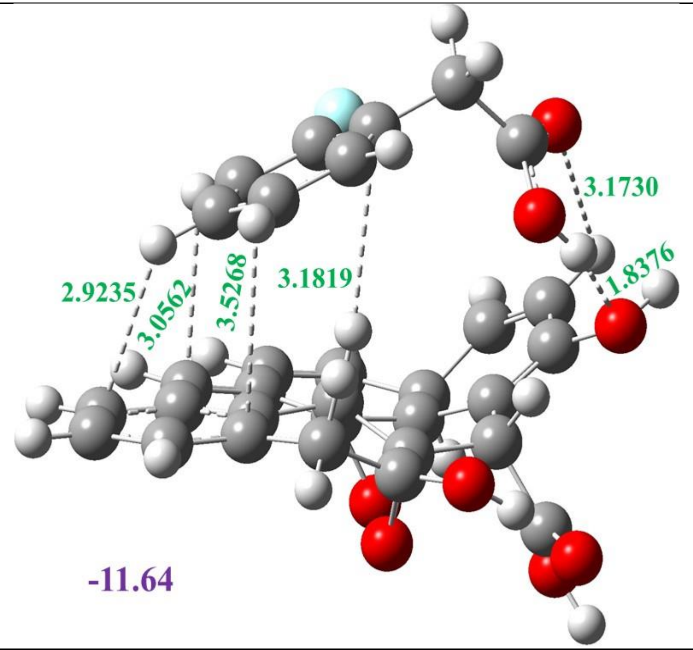

7

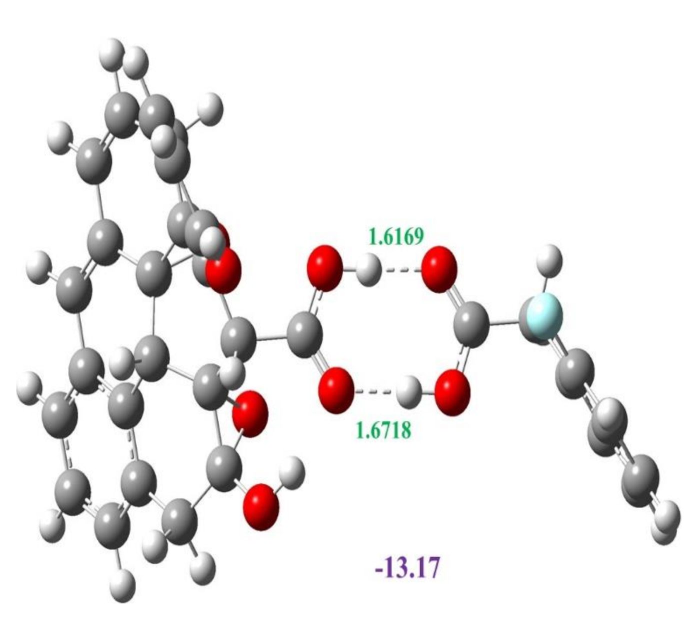

8

GO – neutral pH

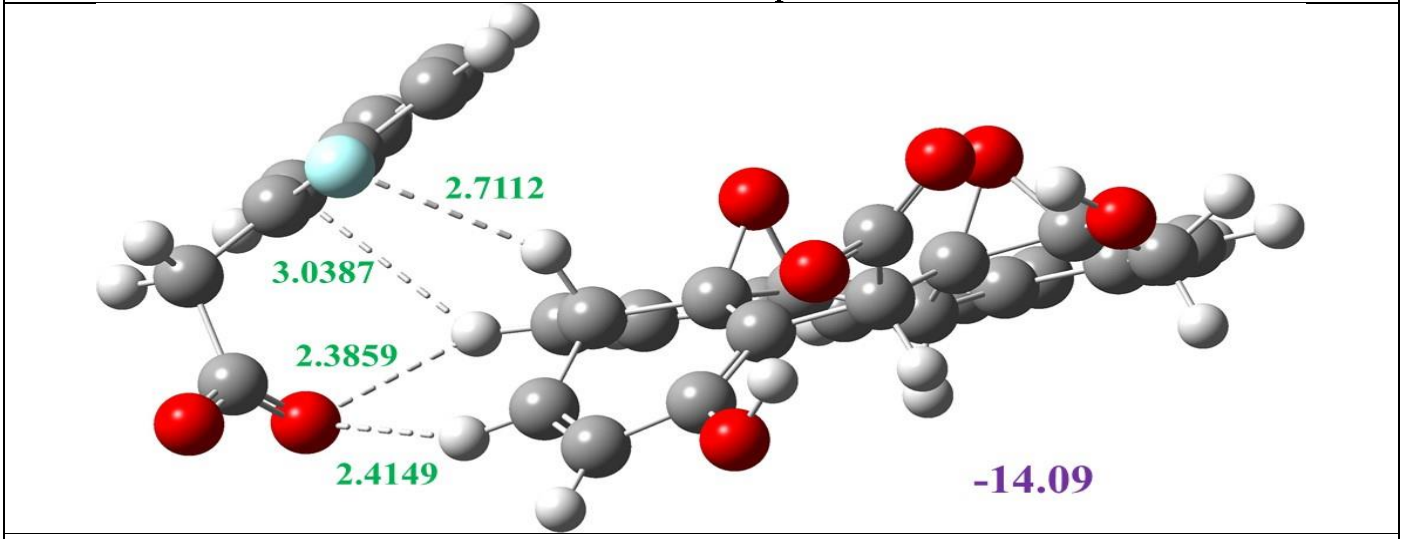

1

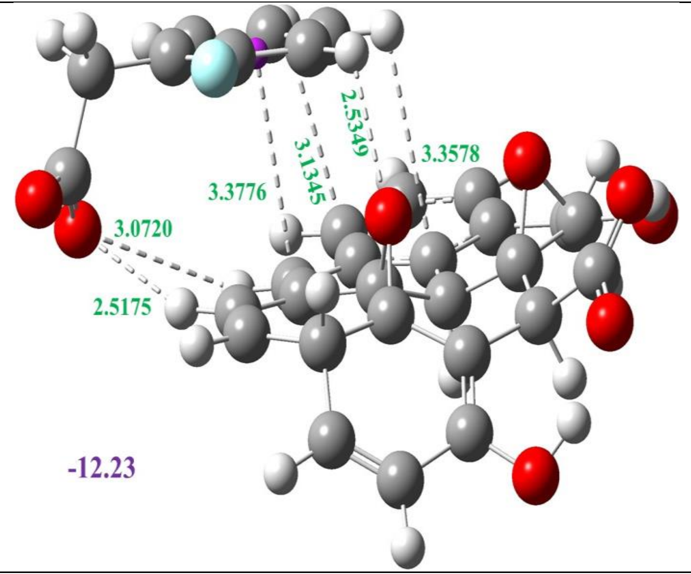
2.5349
3.3578
3.1345
3.3776
3.0720
2.5175
-12.23

1_1

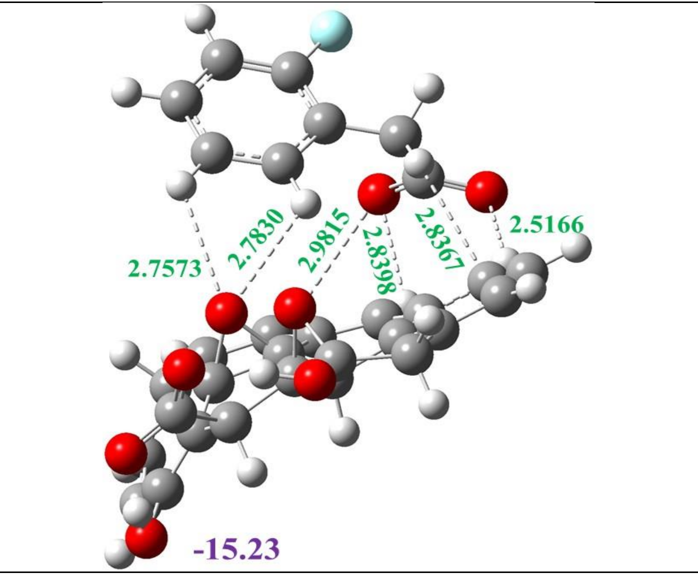
2.7573
2.7830
2.9815
2.8398
2.8367
2.5166
-15.23

2

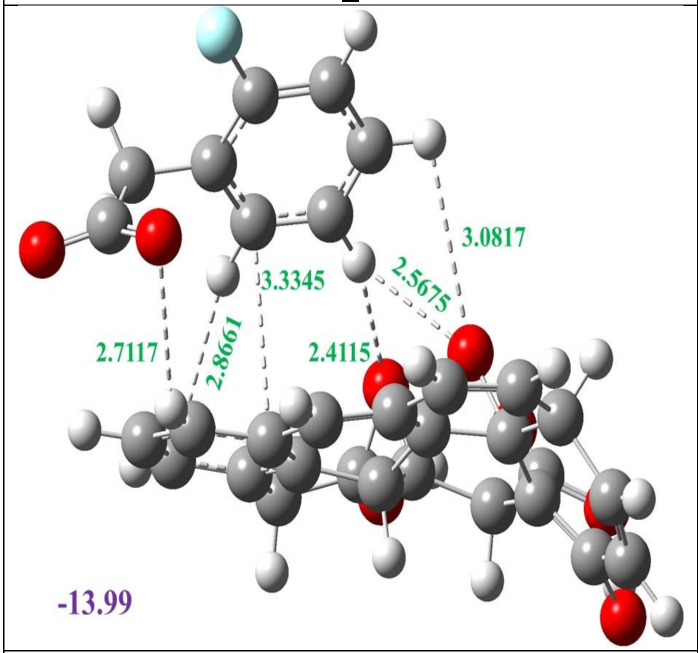
3.0817
3.3345
2.5675
2.7117
2.8661
2.4115
-13.99

3

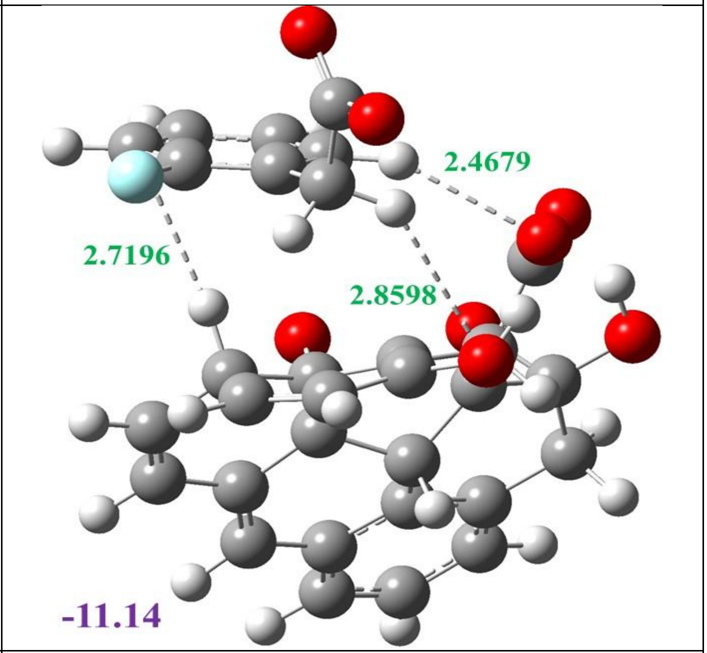
2.4679
2.7196
2.8598
-11.14

4

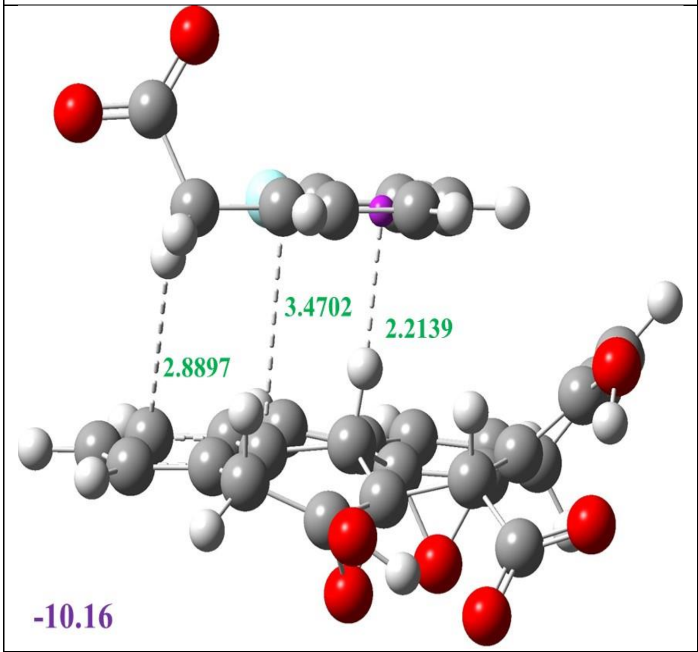
3.4702
2.2139
2.8897
-10.16

5

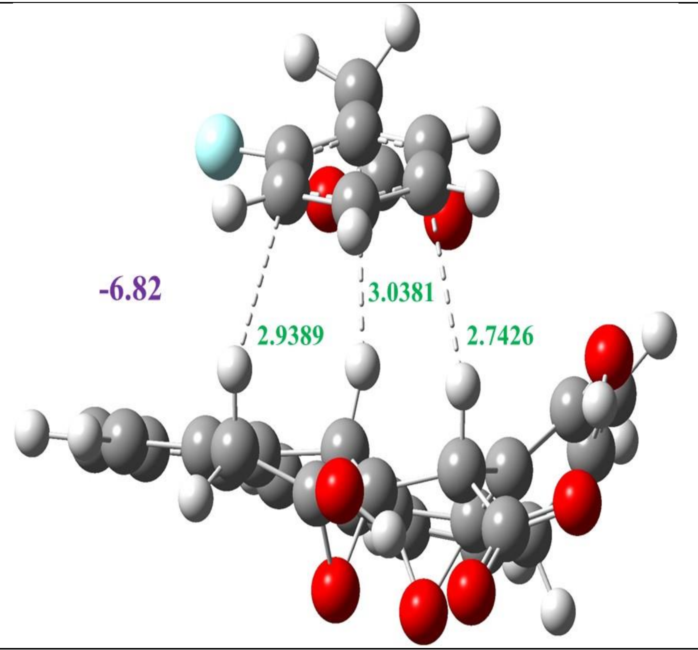
-6.82
3.0381
2.9389
2.7426

6

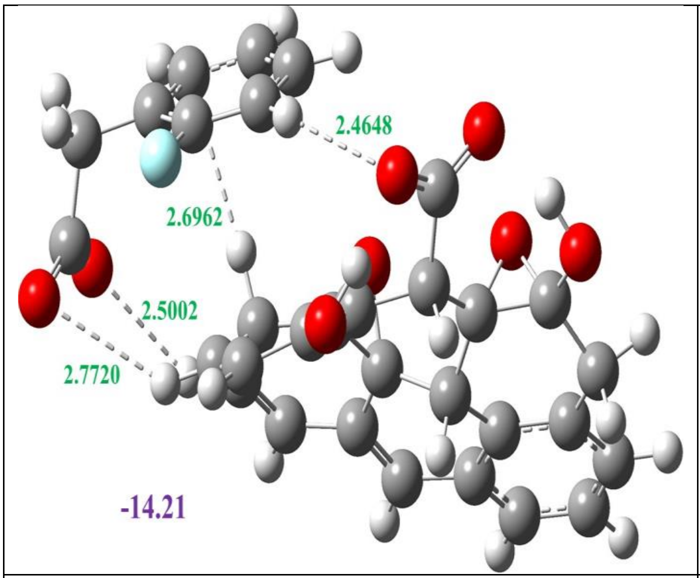
2.4648
2.6962
2.5002
2.7720
-14.21

7

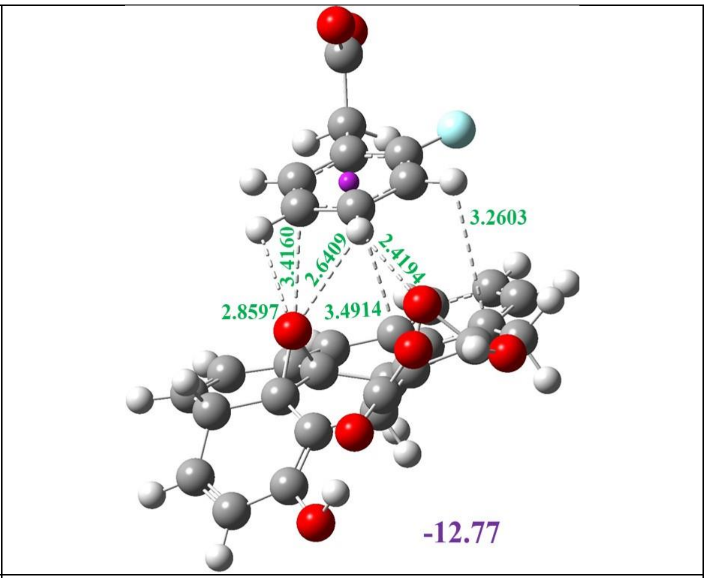
3.2603
3.4160
2.6409
2.4194
2.8597
3.4914
-12.77

8

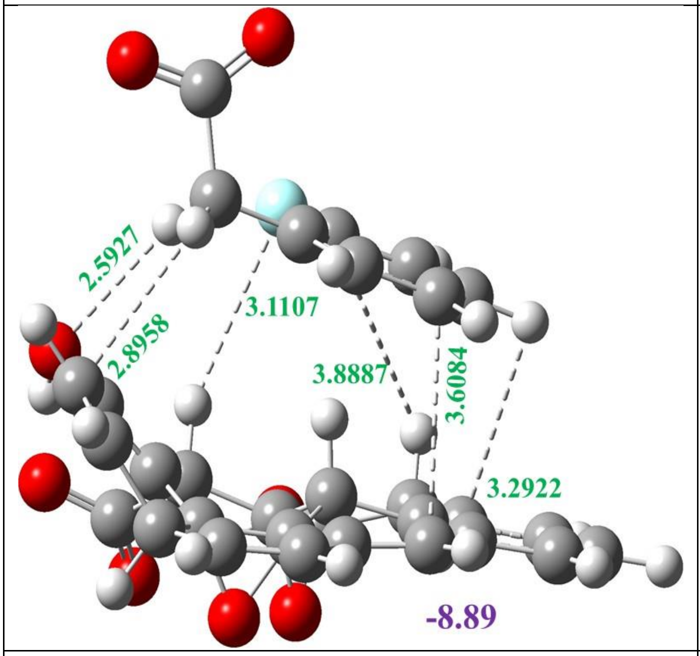
2.5927
2.8958
3.1107
3.8887
3.6084
3.2922
-8.89

9

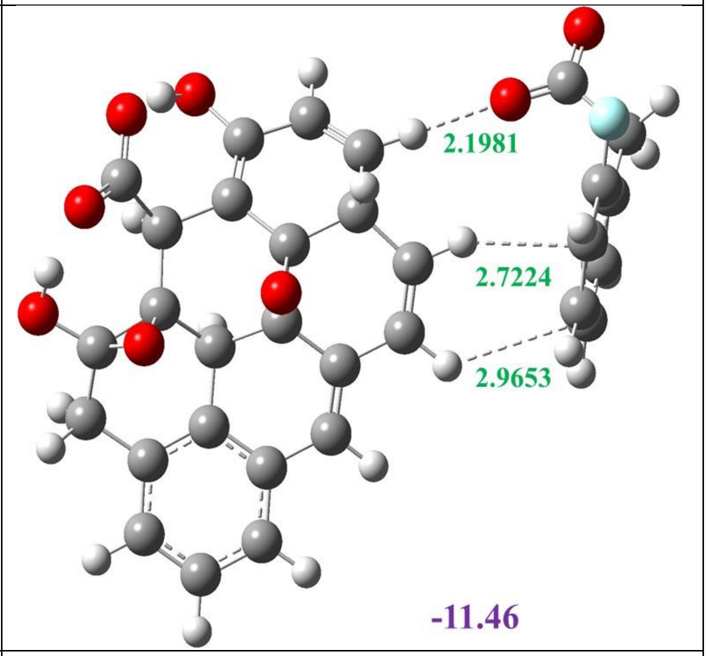
2.1981
2.7224
2.9653
-11.46

10

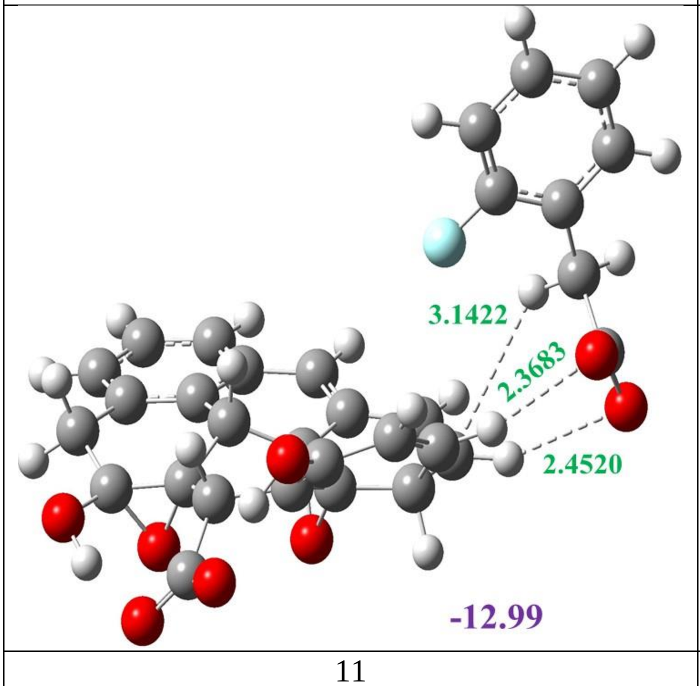
3.1422
2.3683
2.4520
-12.99

11

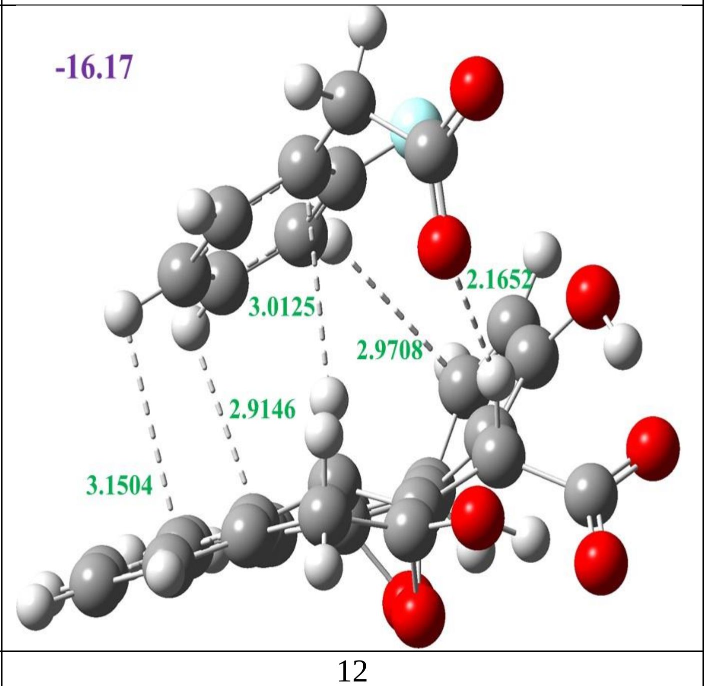
-16.17
2.1652
3.0125
2.9708
2.9146
3.1504

12

rGO – acidic pH

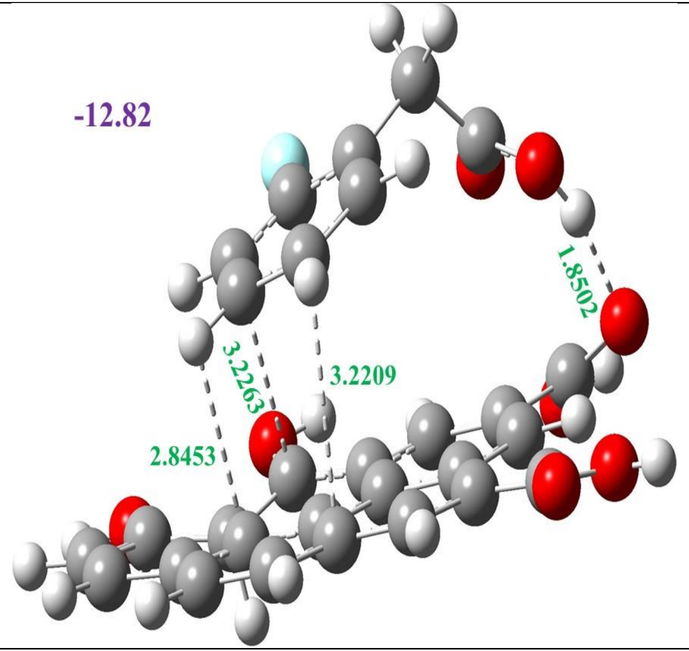

1

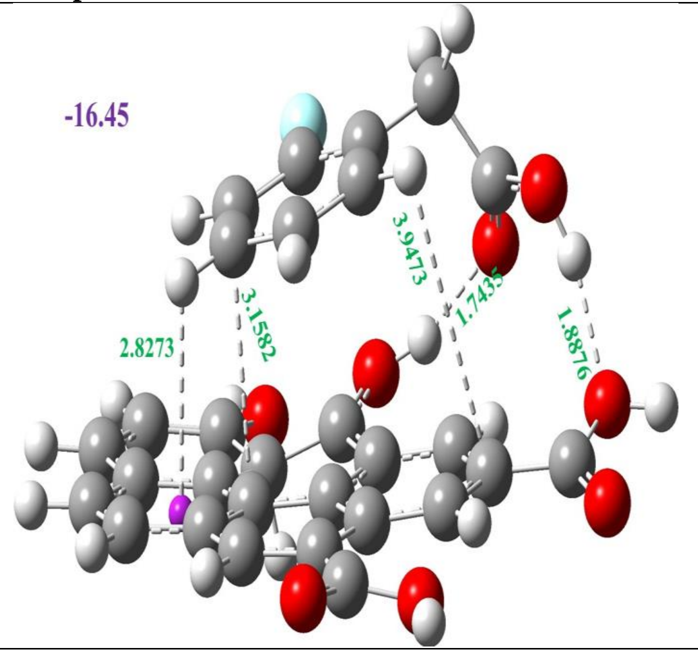

2

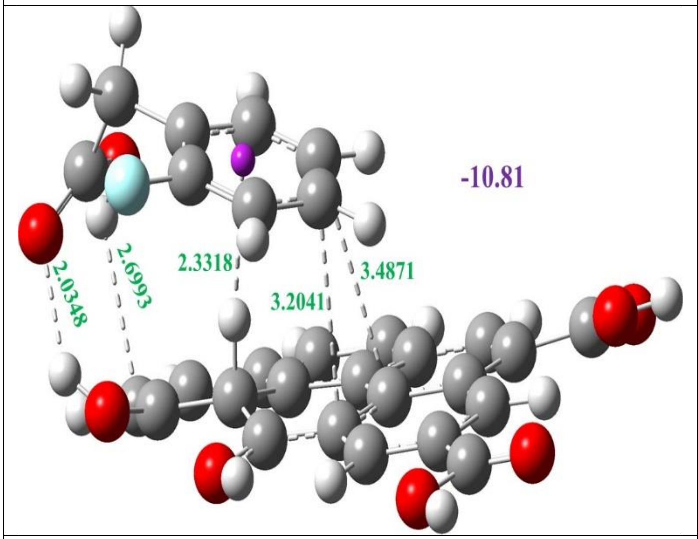

3

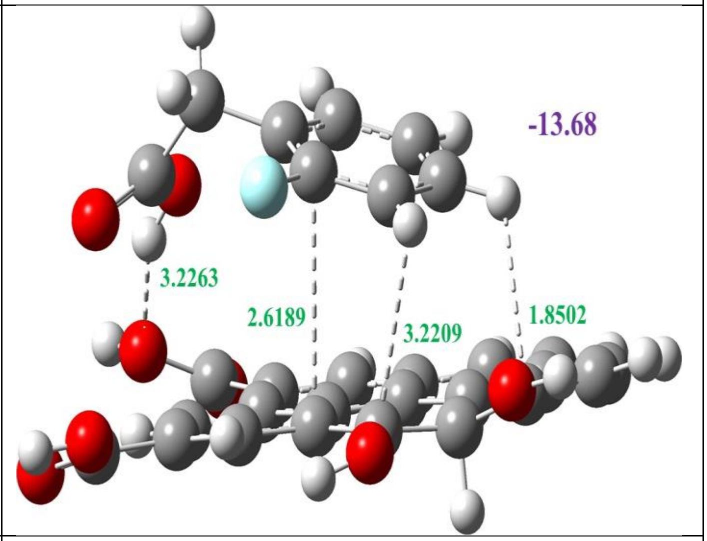

4

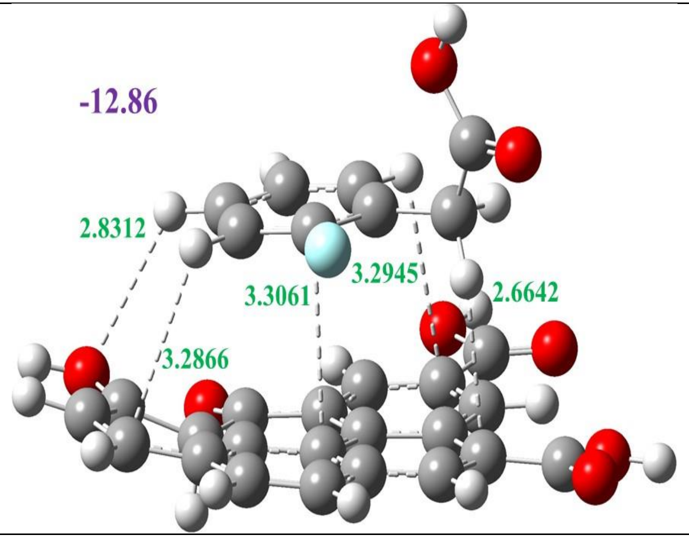

5

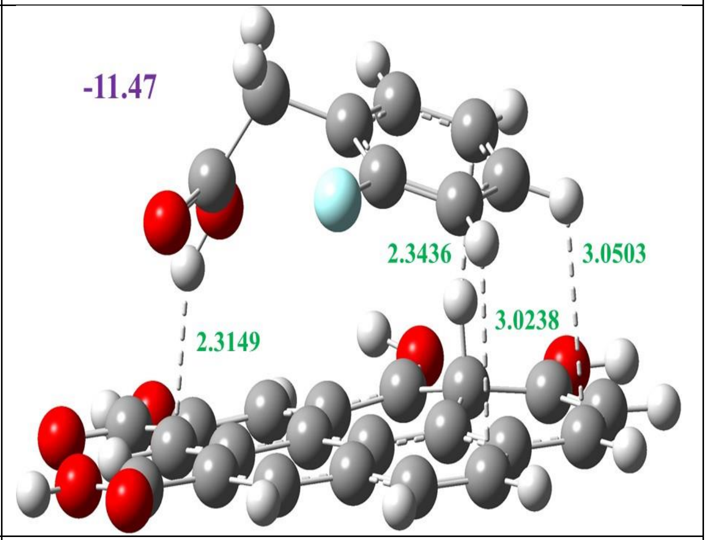

6

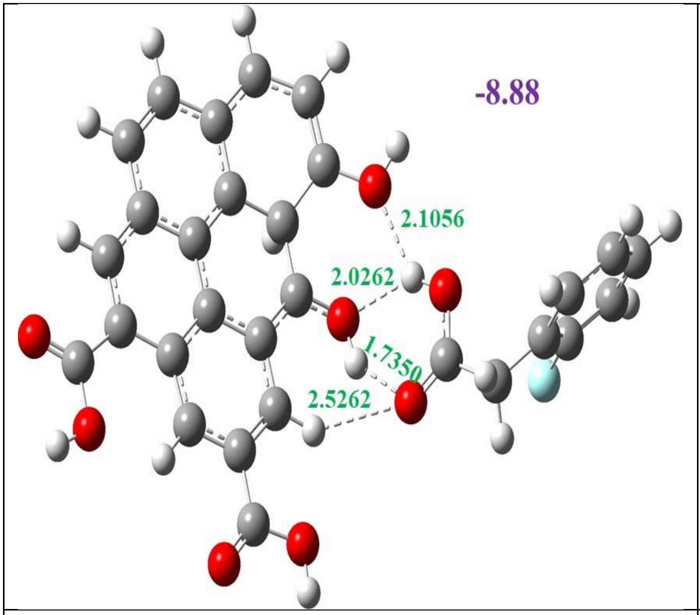	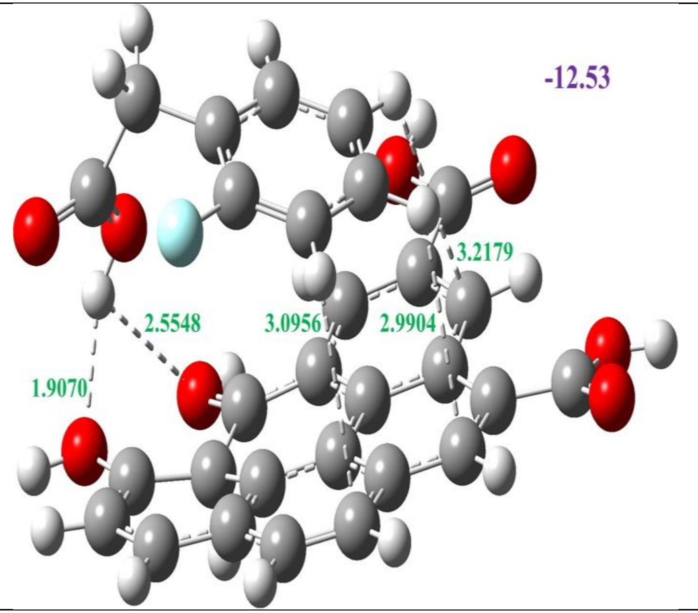
7_1	7_2
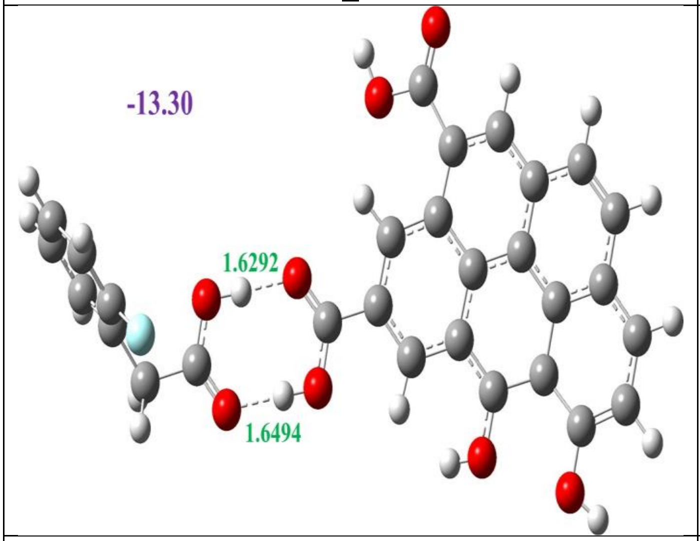	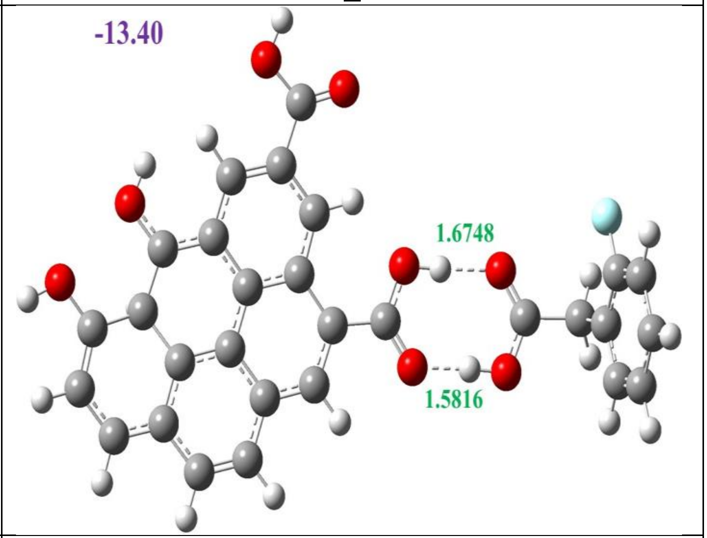
8_1	8_2
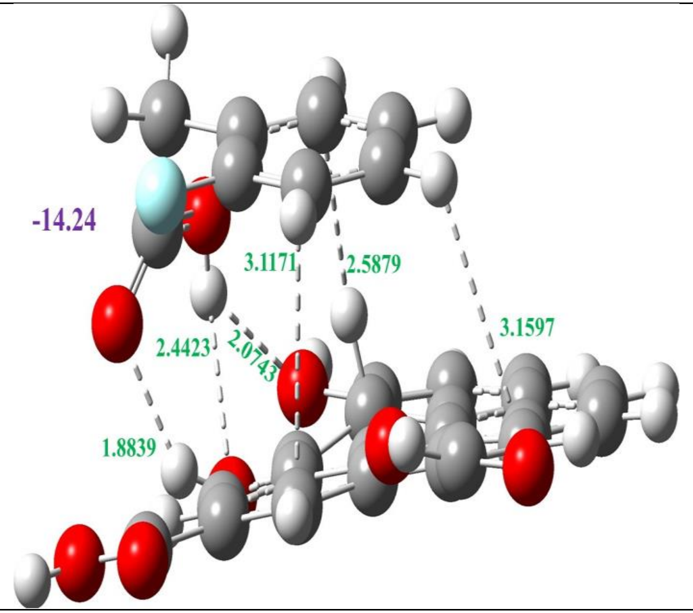	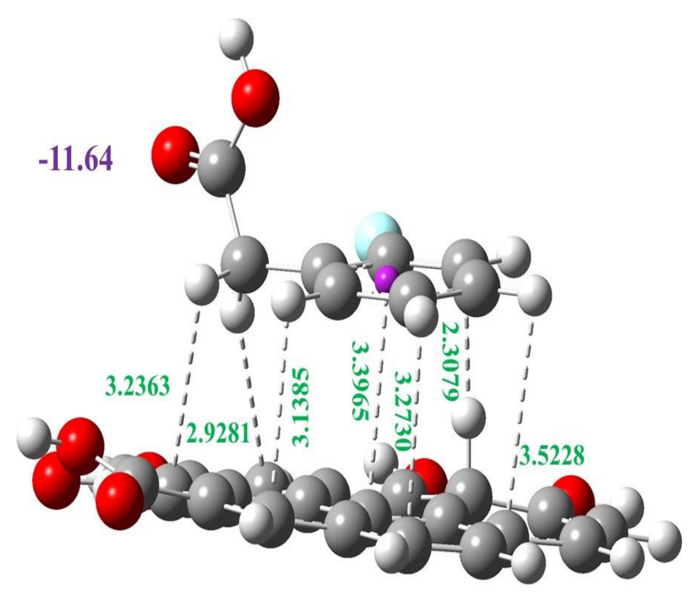
9	10

<table>
<tr><td colspan="2">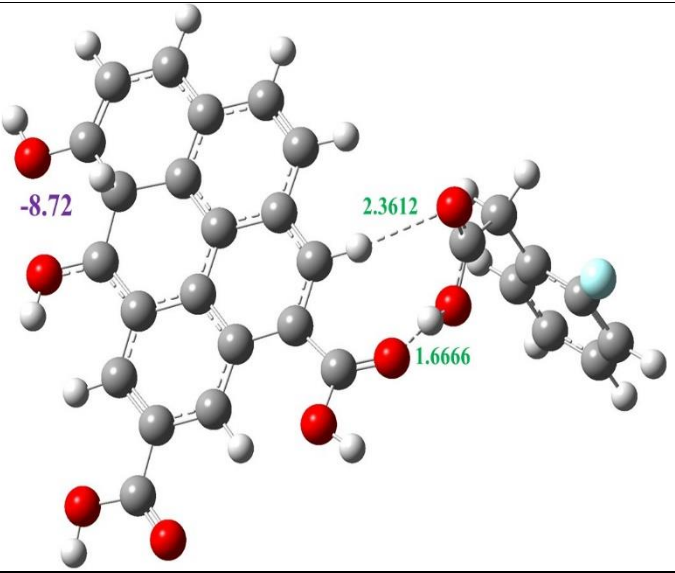
</td></tr>
<tr><td colspan="2">12</td></tr>
<tr><td colspan="2">rGO – neutral pH</td></tr>
<tr><td>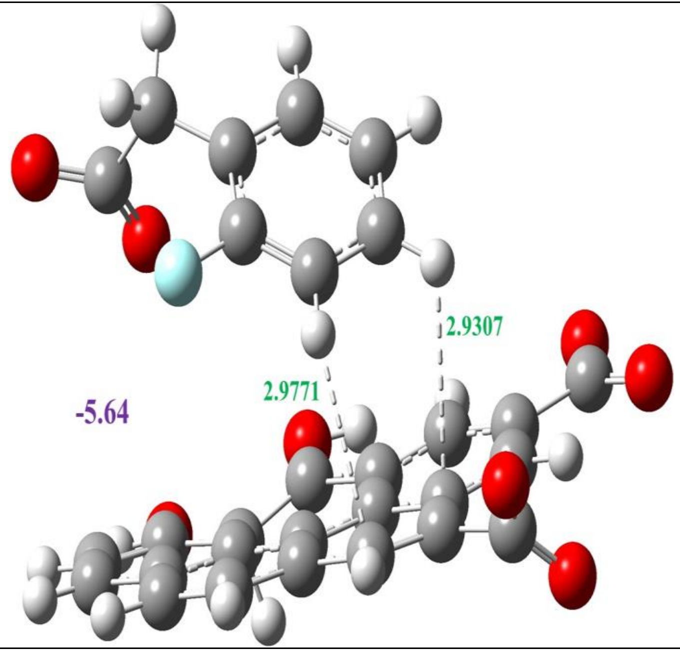
</td><td>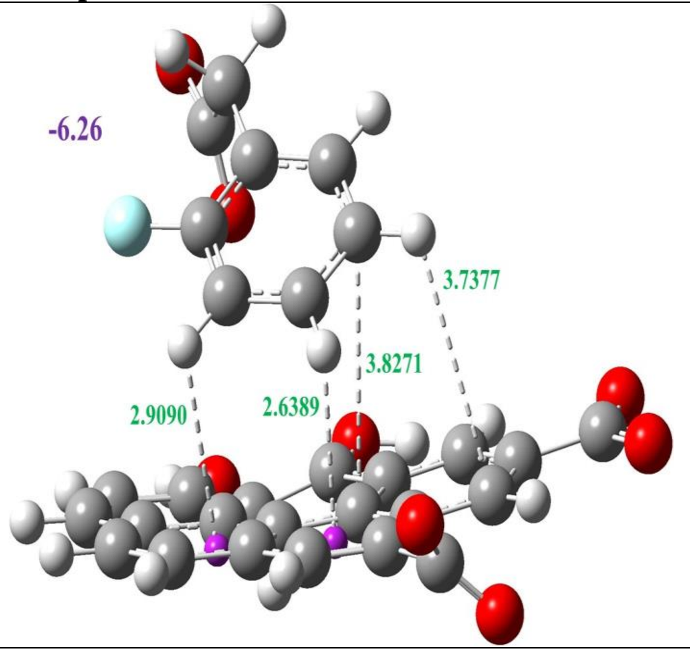
</td></tr>
<tr><td>1</td><td>2</td></tr>
<tr><td>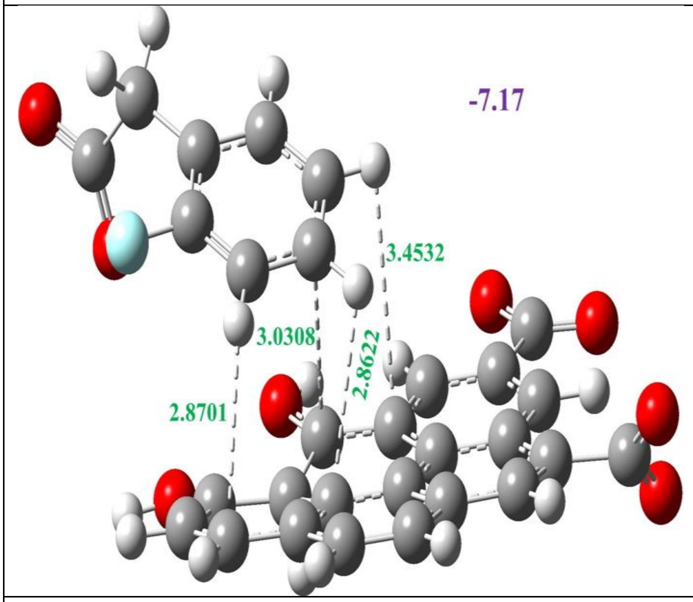
</td><td>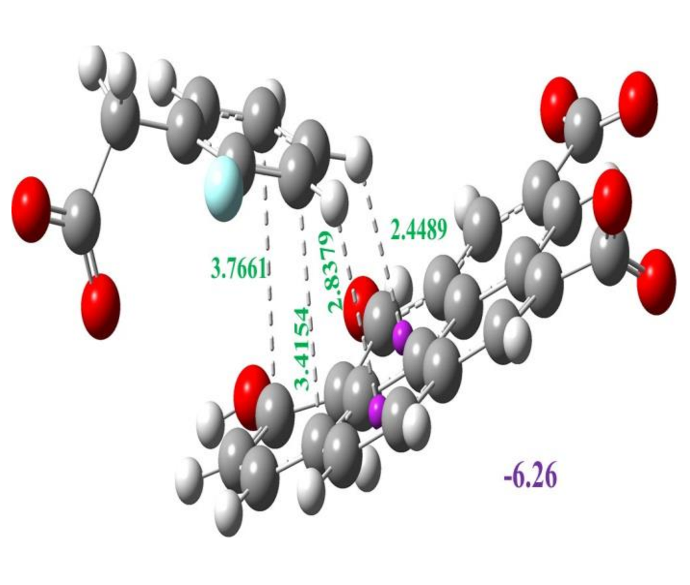
</td></tr>
<tr><td>3_1</td><td>5</td></tr>
</table>

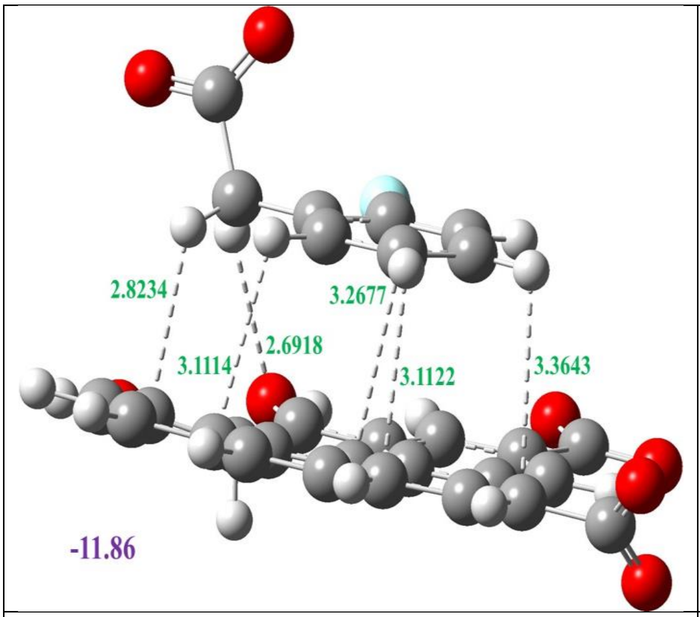
2.8234
3.2677
3.1114
2.6918
3.1122
3.3643
-11.86

6

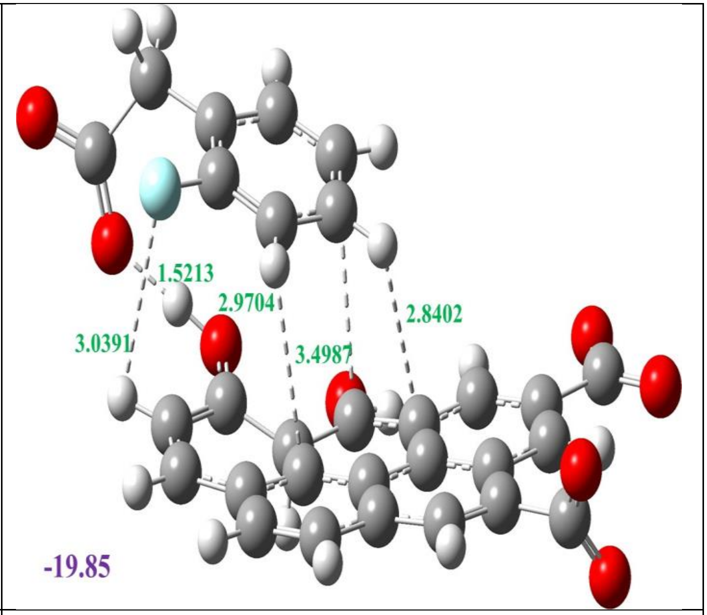
1.5213
2.9704
2.8402
3.0391
3.4987
-19.85

7

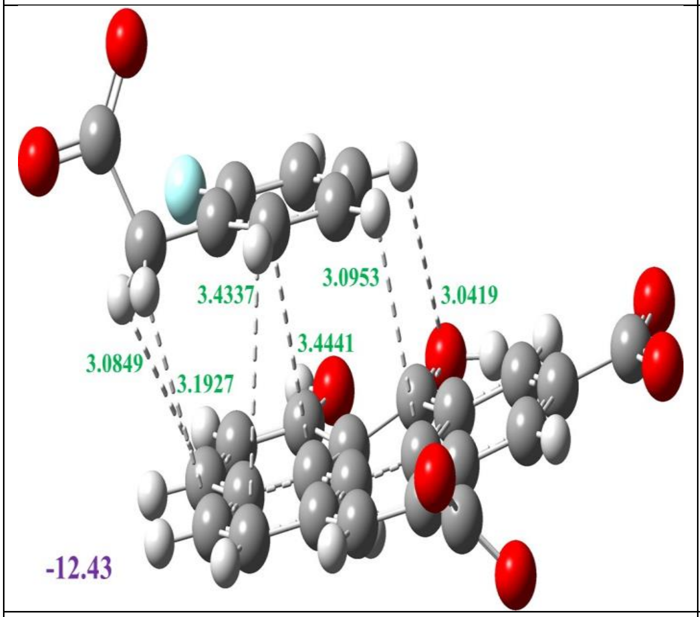
3.4337
3.0953
3.0419
3.4441
3.0849
3.1927
-12.43

8

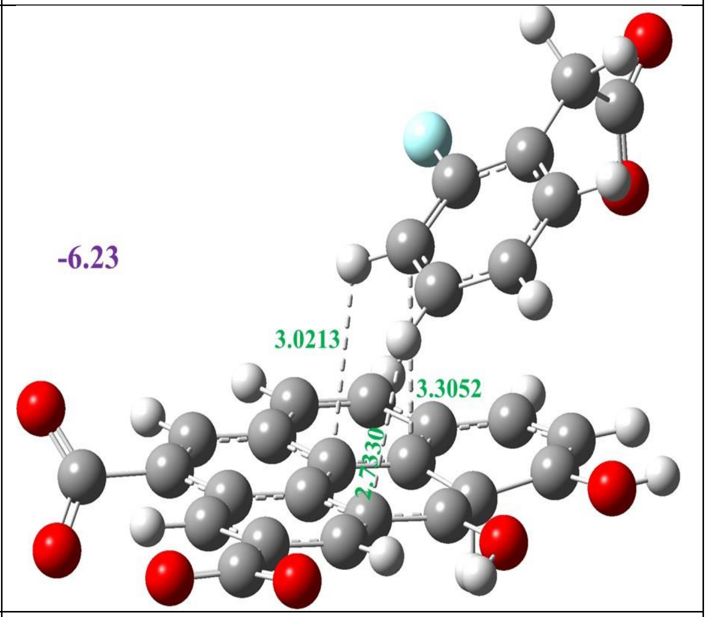
-6.23
3.0213
3.3052

9

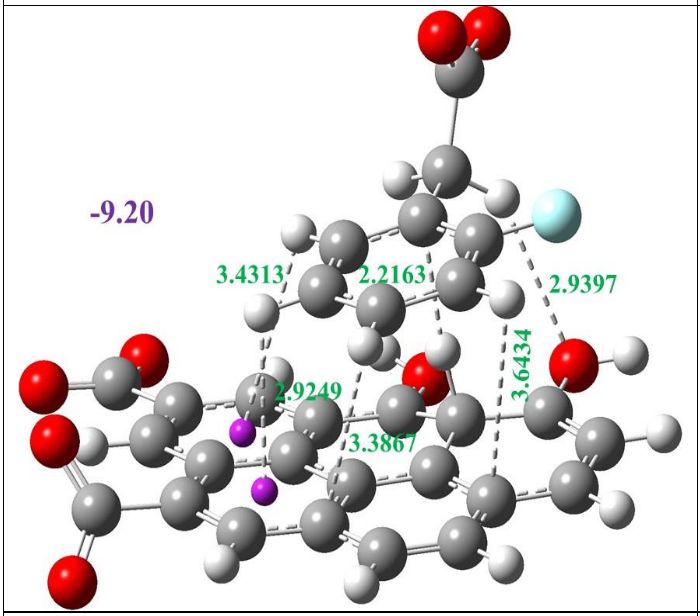
-9.20
3.4313
2.2163
2.9397
2.9249
3.6434
3.3867

10

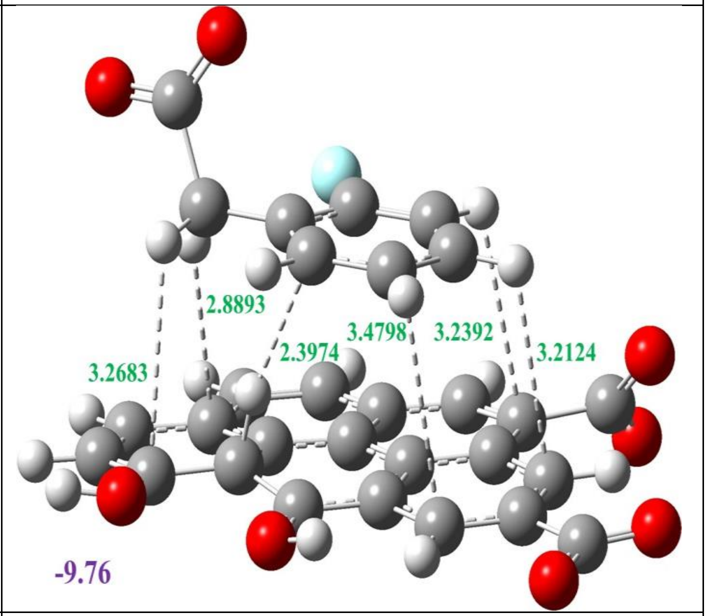
2.8893
3.2683
2.3974
3.4798
3.2392
3.2124
-9.76

11

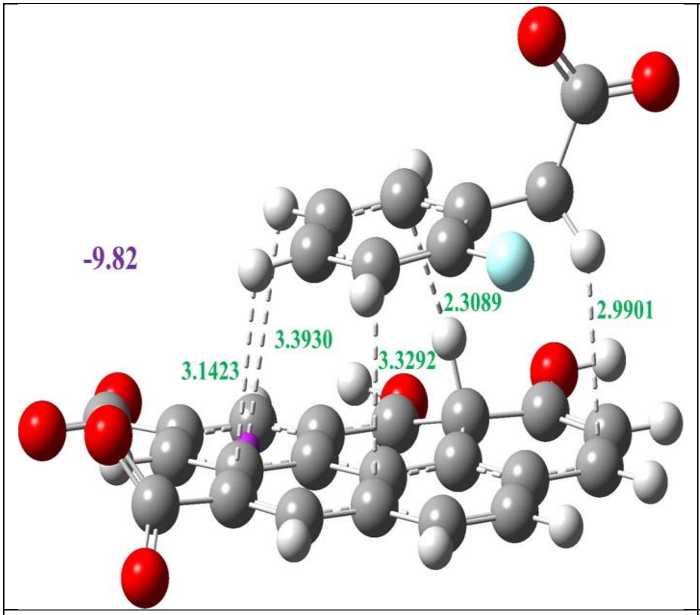

12

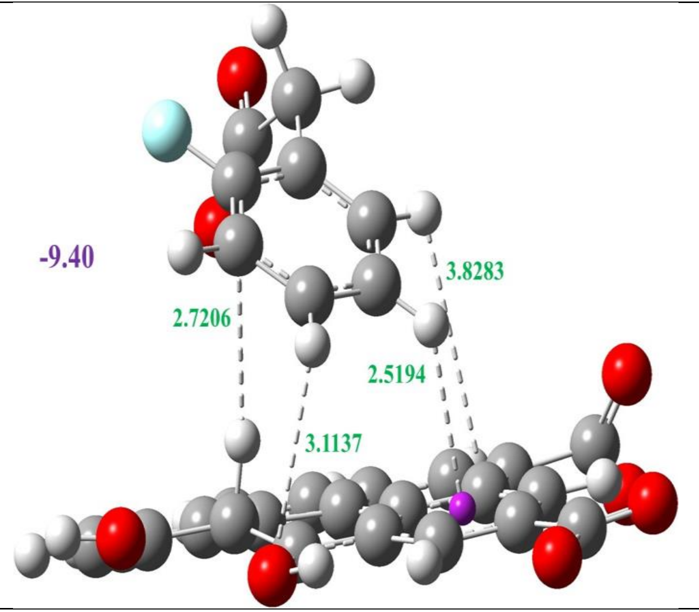

13

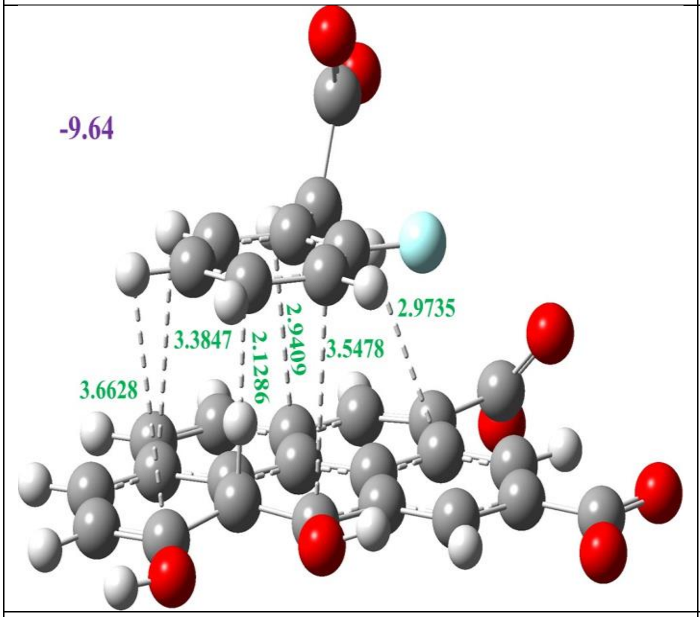

14

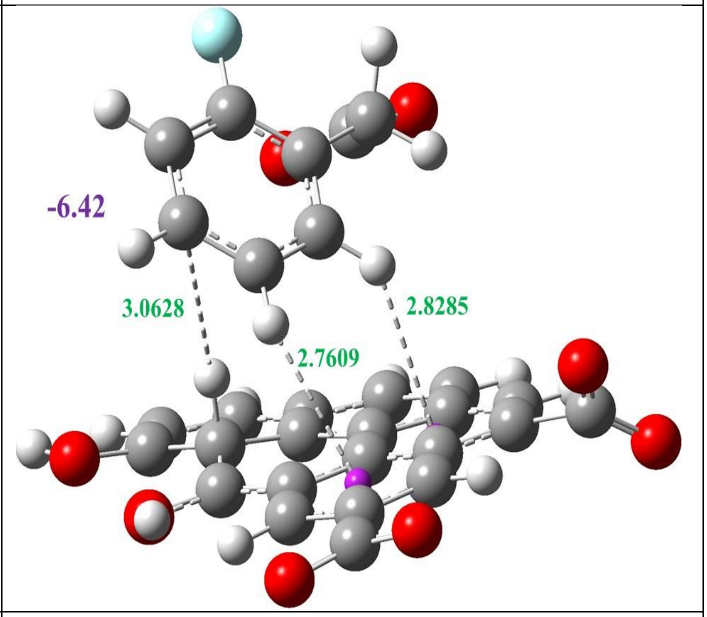

16

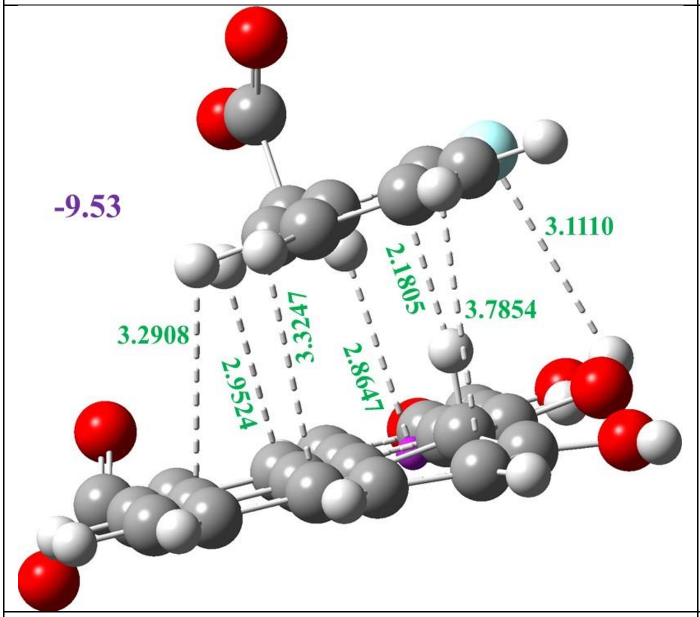

17

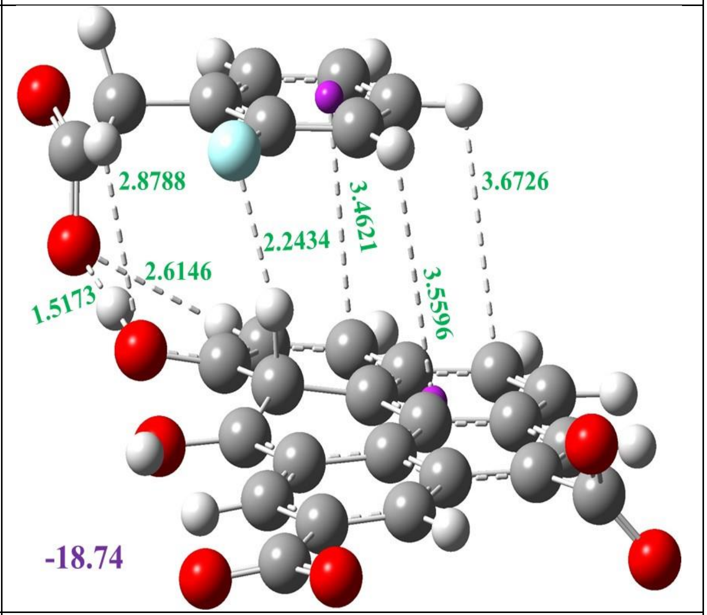

20

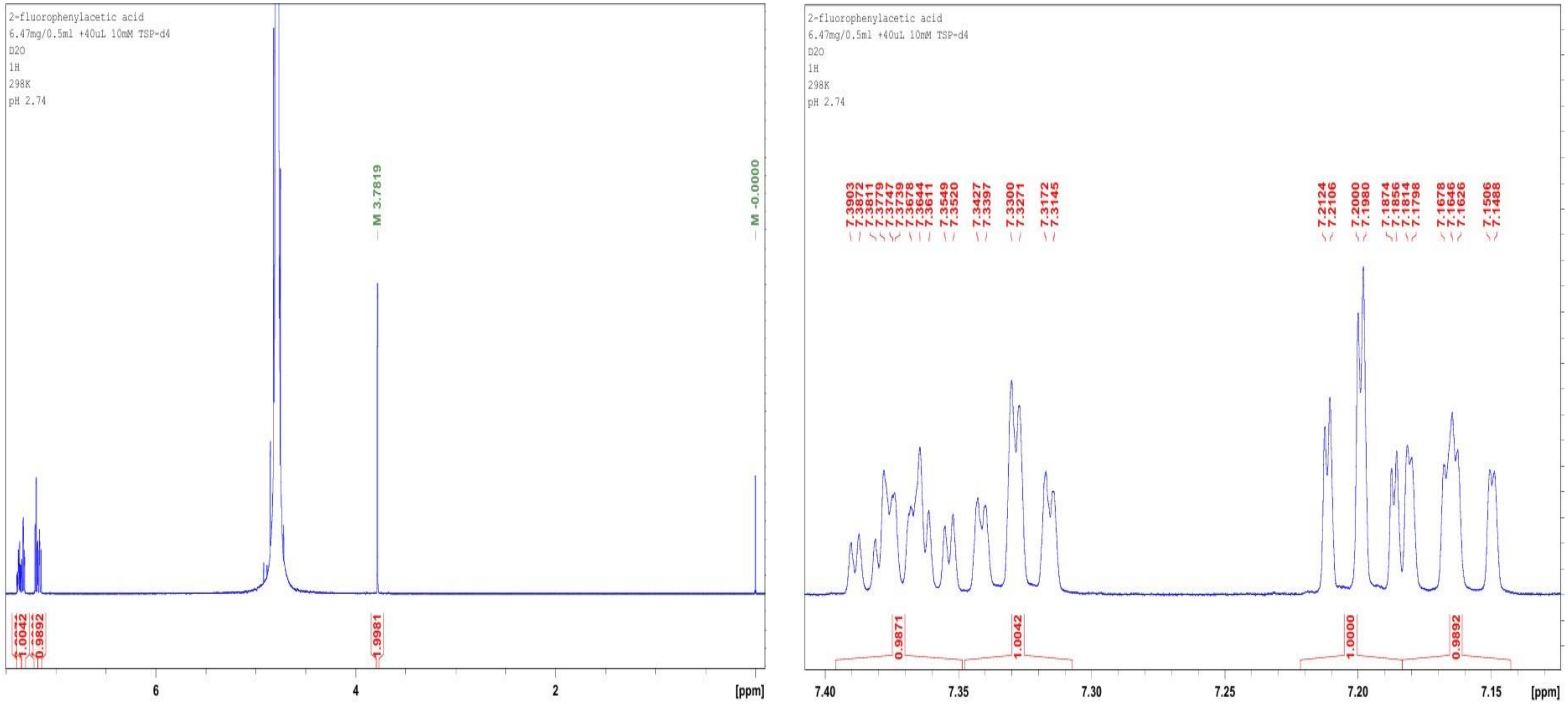

Figure S1. ^{1}H NMR spectrum of 2-fluorophenylacetic acid in acidic environment.

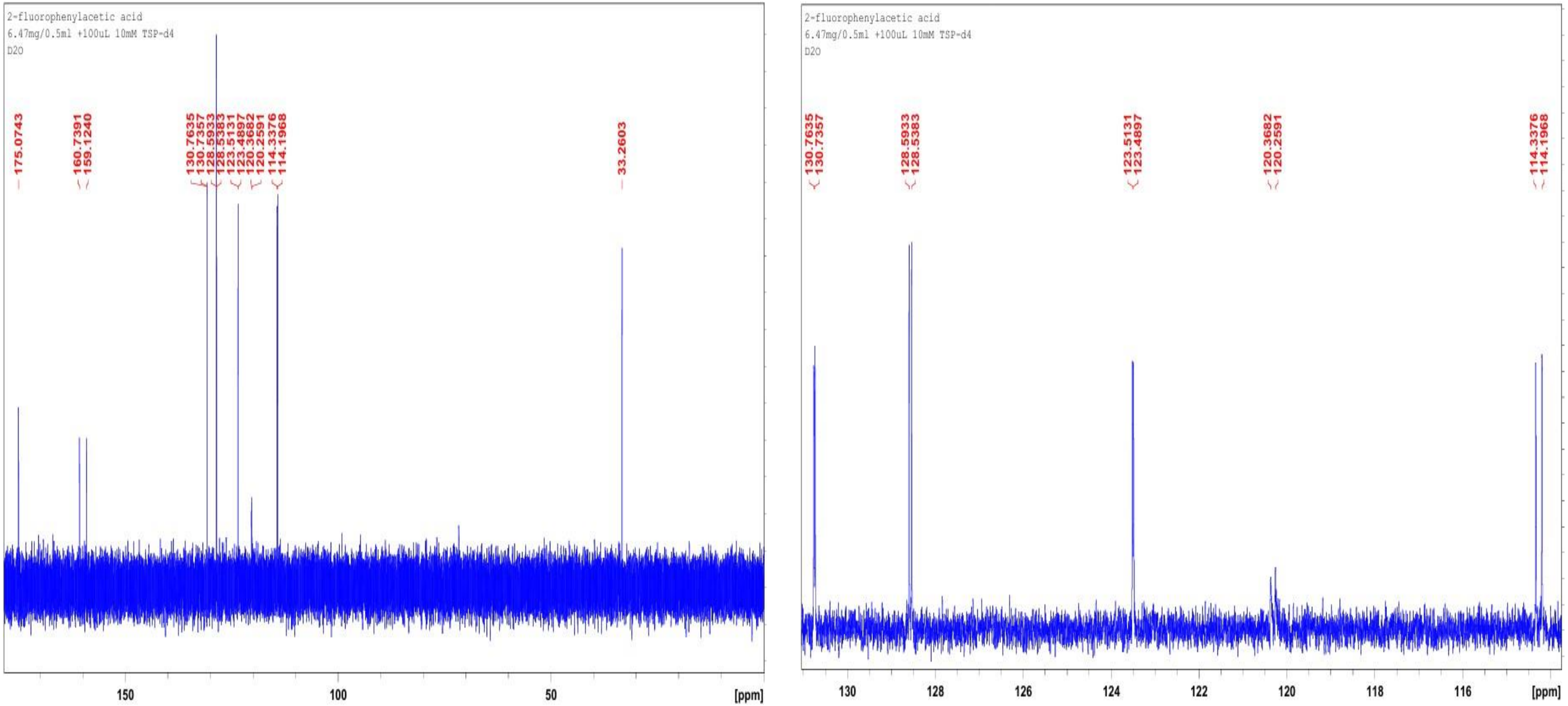

Figure S2. ^{13}C NMR spectrum of 2-fluorophenylacetic acid in acidic environment.

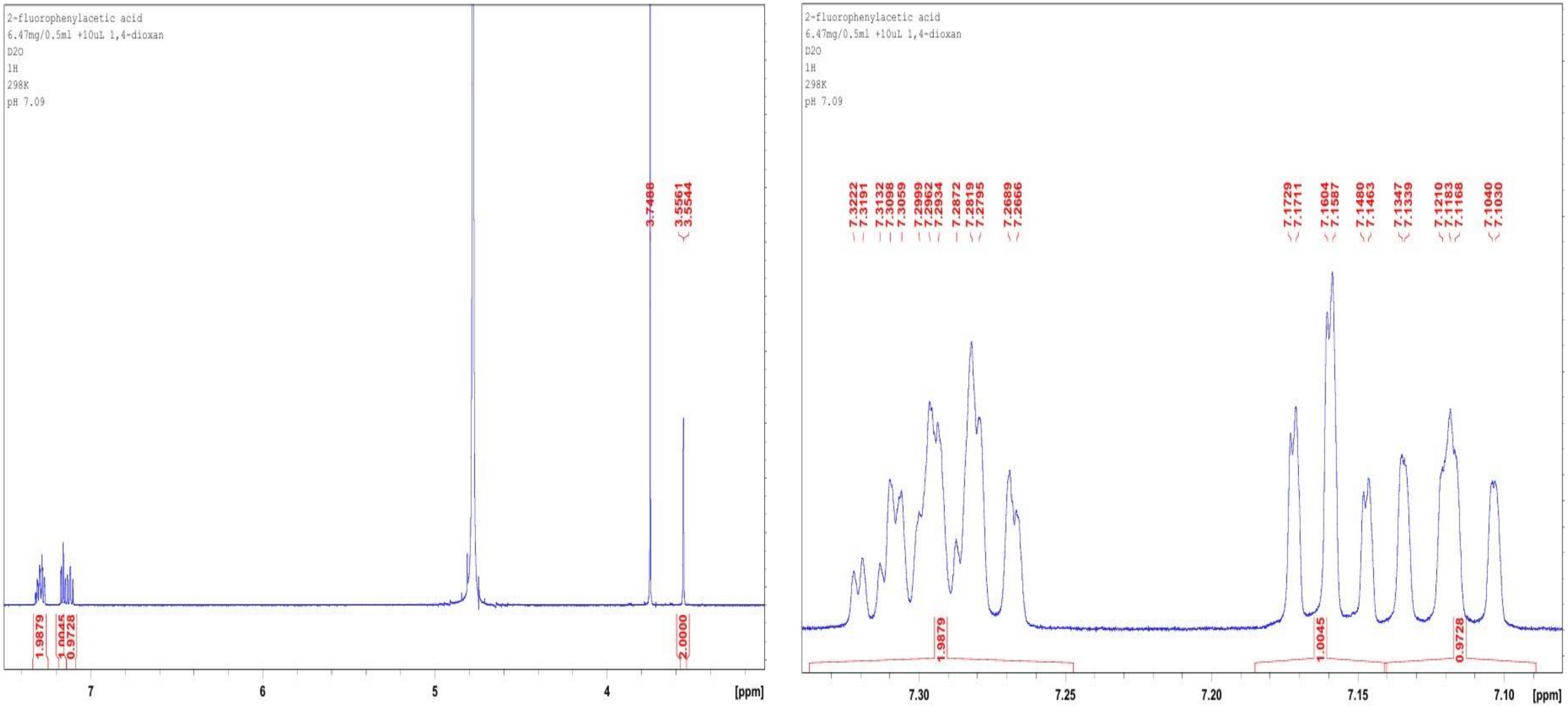

Figure S3. ^{1}H NMR spectrum of 2-fluorophenylacetic acid in neutral environment.

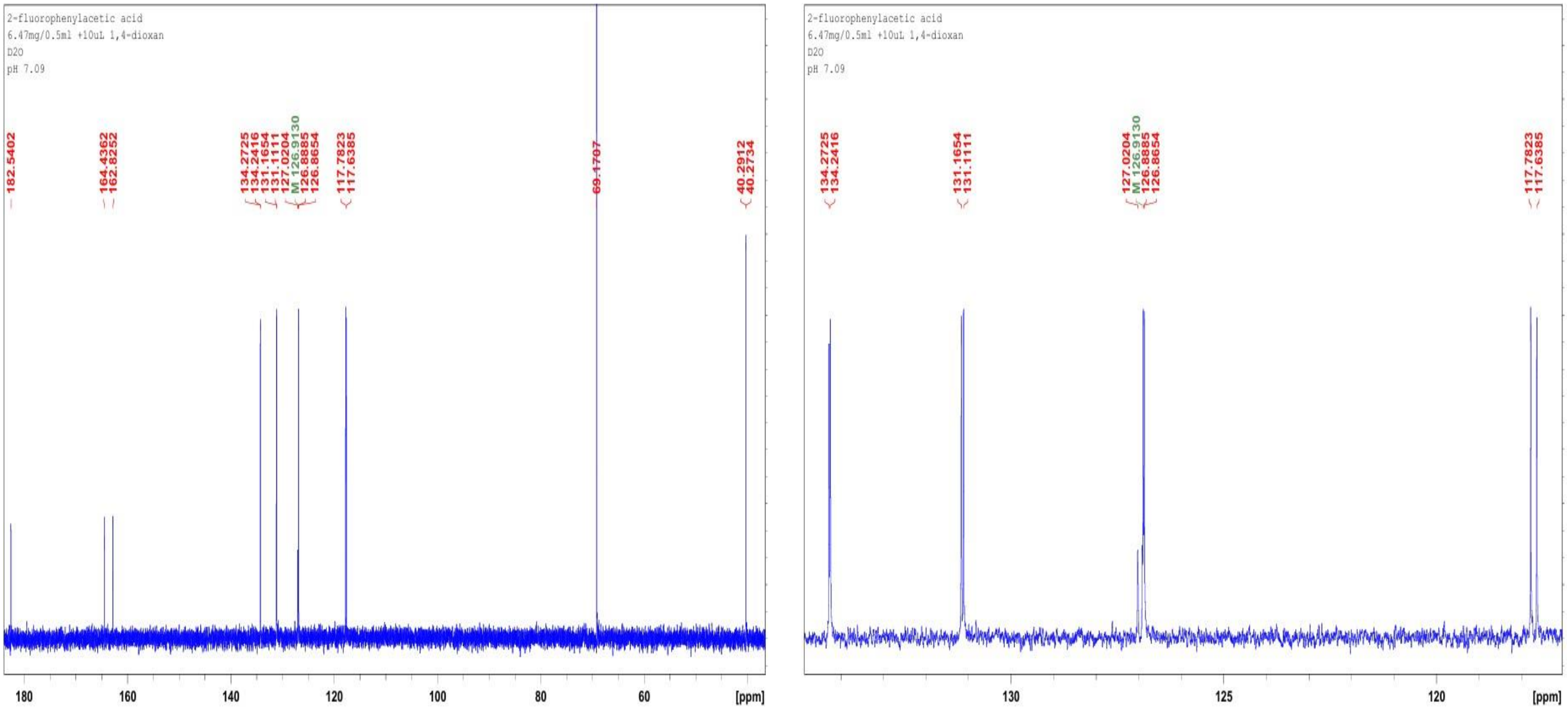

Figure S4. ^{13}C NMR spectrum of 2-fluorophenylacetic acid in neutral environment.

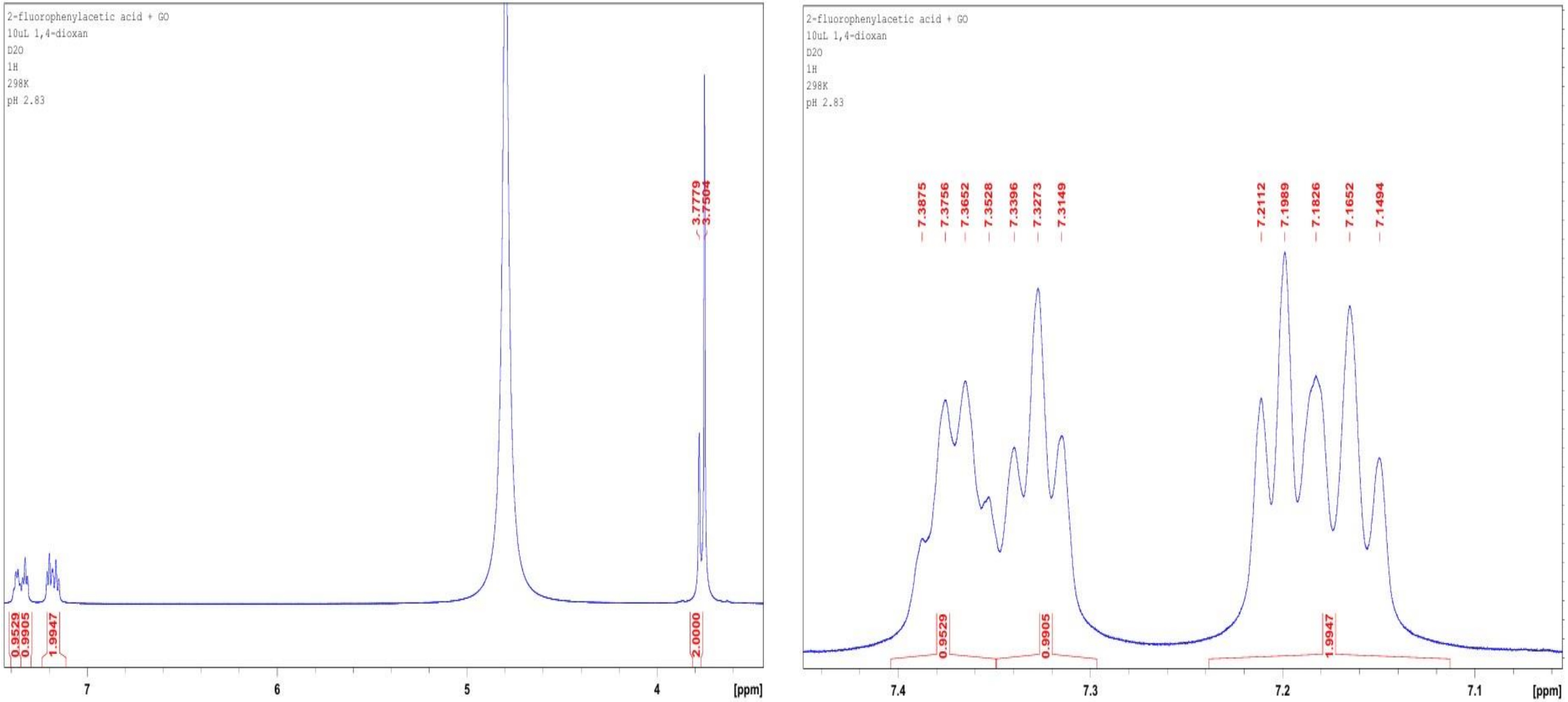

Figure S5. ^{1}H NMR spectrum of 2-fluorophenylacetic acid in acidic environment after GO addition.

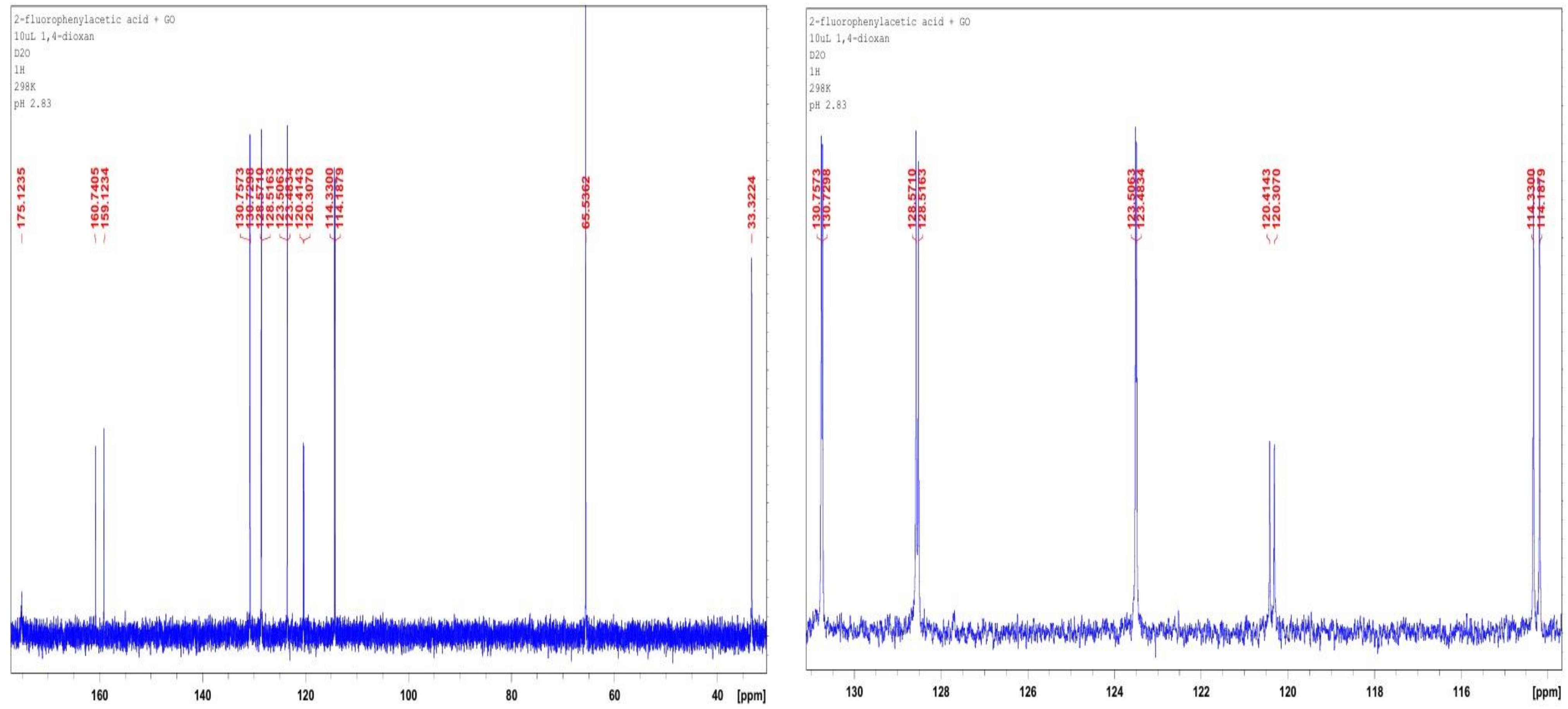

Figure S6. ^{13}C NMR spectrum of 2-fluorophenylacetic acid in acidic environment after GO addition.

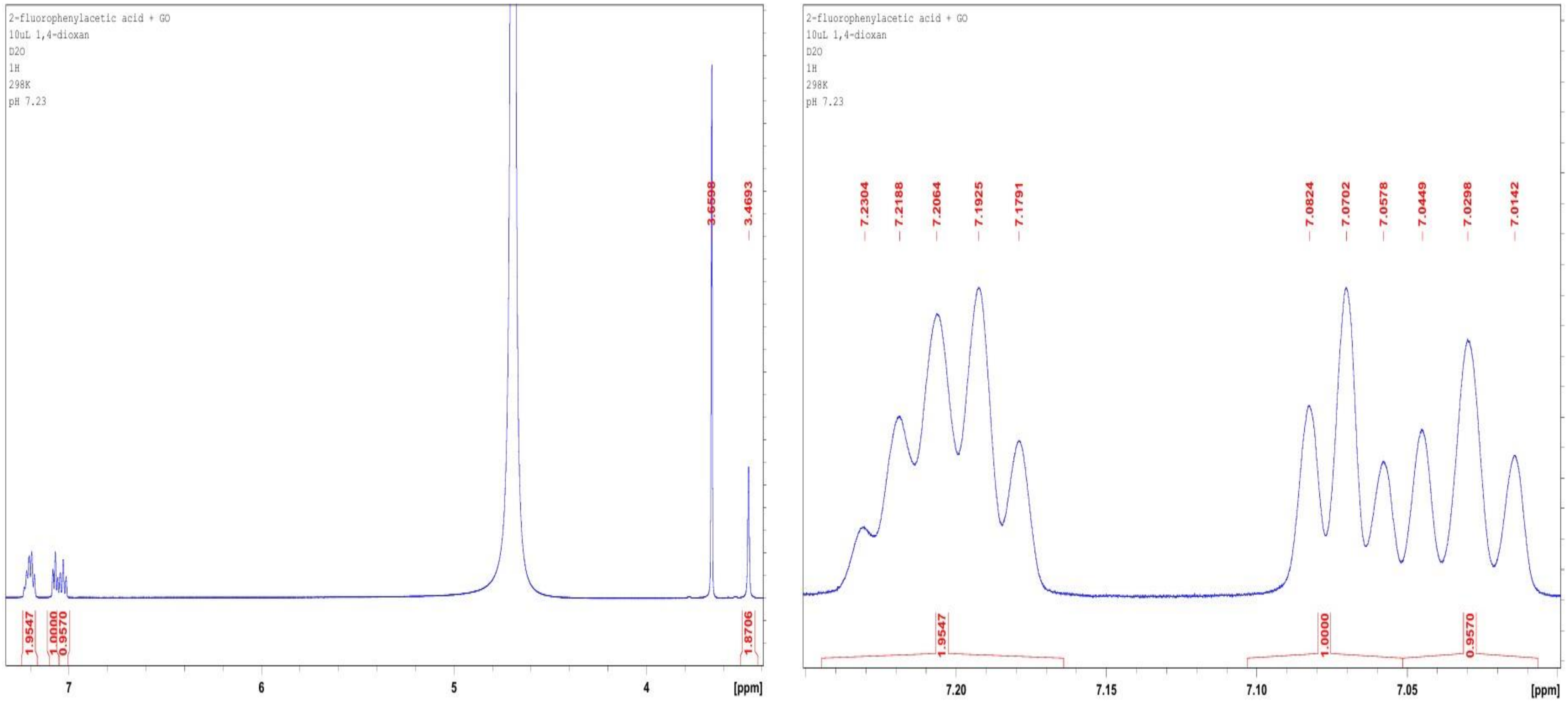

Figure S7. ^{1}H NMR spectrum of 2-fluorophenylacetic acid in neutral environment after GO addition.

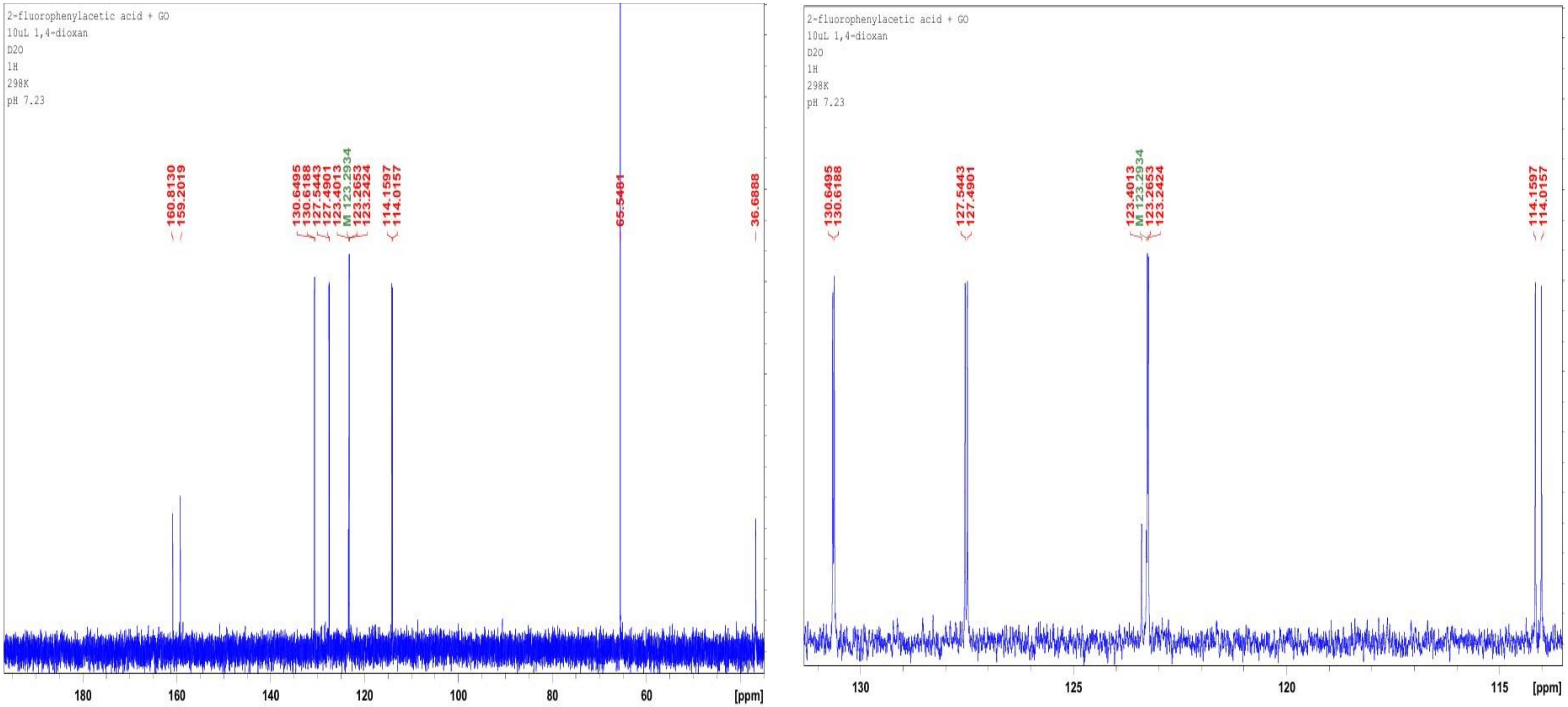

Figure S8. ^{13}C NMR spectrum of 2-fluorophenylacetic acid in neutral environment after GO addition.
